# Supplementary material for: Attitudes and adherence to changes in nutrition and physical activity following surgery for prostate cancer: a qualitative study
Source: BMJ Open. 2022 Jun 29;12(6):e055566. doi: 10.1136/bmjopen-2021-055566 (PMC9244678; doi:10.1136/bmjopen-2021-055566)
Supplement: Supplementary data [file bmjopen-2021-055566supp001.pdf]

Patient Interview Topic Guide

PrEvENT: End of trial evaluation interview

Introduction

1. Can you tell me a little about your involvement in the trial?
   a) What arm were you allocated to?
   b) What exactly were you asked to do?

Intervention specifics

2. How would you describe your overall experience of taking part in the intervention?

3. What were the positives of…(intervention arm)

4. Were there any negative elements of…(intervention arm)

5. Would you change any elements of your intervention arm?
   a. Brisk walking for longer / shorter
   b. Add other supplements / take supplements less often
   c. Add more or less changes to diet
      i. If so, which / how?

6. How did you find the instructions you were given about your intervention arm and daily monitoring instructions?
   a. Could you suggest improvements to the instructions?

7. We asked you to make changes to your behaviour approximately 6 weeks after surgery; do you think this was too soon / not soon enough?
   a. What do you think would be the ideal time to make changes to behaviour?

8. How did you find wearing the PA monitoring tool?
   a. Were you able to wear it all of the time? If not, why not?
   b. When were you not able to wear the PA monitoring tool? Why not?
   c. What were barriers to wearing it?
   d. What were benefits to wearing it?
   e. What made it easier?
   f. How did you find the instructions that you were provided with for the monitor?
   g. Could you suggest improvements to the instructions?

9. If we had asked you to stay in the study for longer eg. carrying out the intervention, or completing questionnaires every 6 months, how would you feel about that?
   a. What would make that easier?

Behaviour change

10. How easy or difficult did you find making changes to your behaviour?
    a. Do you think this would have been different in a different arm? How?
11. Do you think you would be able to continue with these changes for a longer duration of time?
12. How easy or difficult did you find it to remember to carry out the intervention changes?
    a. Why was this?
    b. How did you remember / what advice could you give others to remember?
13. Did you talk to friends / family about the trial and the changes we asked you to make?
    a. How did they respond?
    b. Were they supportive? Critical? Sceptical?
    c. How did that affect you and your behaviours?
14. Have you seen or felt any benefits / negative effects from the changes we asked you to make?
15. Did you see any weight differences due to the change in diet or physical activity?
    a. Would that have made a difference to you?
16. Would you say you have made any lasting changes to your behaviour as a result of the research?
    a. What behavioural changes have you made?
    b. Why did you make these changes?
    c. Why do you plan to continue with these changes?
17. Has participating in the research made you think about your behaviours differently at all?
18. Do you plan to continue with the changes you made?
    a. If so, which? Why? Why not?
19. What are your opinions about the associations between diet, physical activity and cancer?
    a. What about the link with obesity?
    b. Would that make you reconsider your behaviours? What would make you reconsider them?

Trial logistics / contact / nurse appointments

20. How would you describe your experience of the trial form a logistic perspective?
    a. How the appointments were made?
    b. Ease of attending appointments?
    c. Length of appointments?

21. How did you find your research clinic appointments with the research nurse?
    a. Were these enjoyable?
    b. Did these affect your motivation to continue with the research?
    c. Were you provided with all of the information that you required?

22. How did you find the regular contact / reminders by the research nurse and research team?
    a. Which method did you find the most useful? Why?
    b. Did they affect your motivation?
    c. Do you think they helped you to remember / continue with the trial?

Overall experience

23. How would you sum up your overall experience of taking part in the trial?

24. How would you improve or change the trial to make it better for future participants?

25. Was participating different to what you had expected?
    a. In a positive / negative way?
    b. What had you expected?
    c. Had you participated in research prior to this?

26. Which elements of the trial did you enjoy the most?

27. Which elements of the trial did you not enjoy? Or enjoy the least?

28. Based on your experience in this trial, would you participate in research again?
    a. If so why/if not why not?

29. Final question, what made you agree to take part in the first place?

30. Is there anything further you wish to add?
