# Supplementary material for: Attitudes and adherence to changes in nutrition and physical activity following surgery for prostate cancer: a qualitative study
Source: BMJ Open. 2022 Jun 29;12(6):e055566. doi: 10.1136/bmjopen-2021-055566 (PMC9244678; doi:10.1136/bmjopen-2021-055566)
Supplement: Author's manuscript [file bmjopen-2021-055566.draft_revisions.pdf]

BMJ Open is committed to open peer review. As part of this commitment we make the peer review history of every article we publish publicly available.

When an article is published we post the peer reviewers' comments and the authors' responses online. We also post the versions of the paper that were used during peer review. These are the versions that the peer review comments apply to.

The versions of the paper that follow are the versions that were submitted during the peer review process. They are not the versions of record or the final published versions. They should not be cited or distributed as the published version of this manuscript.

BMJ Open is an open access journal and the full, final, typeset and author-corrected version of record of the manuscript is available on our site with no access controls, subscription charges or pay-per-view fees (http://bmjopen.bmj.com).

If you have any questions on BMJ Open's open peer review process please email info.bmjopen@bmj.com

BMJ Open

ATTITUDES AND ADHERENCE TO CHANGES IN NUTRITION AND PHYSICAL ACTIVITY FOLLOWING SURGERY FOR PROSTATE CANCER: A QUALITATIVE STUDY

Journal:	*BMJ Open*
Manuscript ID	bmjopen-2021-055566
Article Type:	Original research
Date Submitted by the Author:	20-Jul-2021

Complete List of Authors:	Robles, Luke; University of Bristol, National Institute for Health Research Bristol Biomedical Research Centre, University Hospitals Bristol and Weston NHS Foundation Trust and University of Bristol<br>Shingler, Ellie; University of Bristol, National Institute for Health Research Bristol Biomedical Research Centre, University Hospitals Bristol and Weston NHS Foundation Trust and University of Bristol<br>McGeagh, Lucy ; Oxford Brookes University, Supportive Cancer Care Research Group, Faculty of Health and Life Sciences, Oxford Institute of Nursing, Midwifery and Allied Health Research; University of Bristol, National Institute for Health Research Bristol Biomedical Research Centre, University Hospitals Bristol and Weston NHS Foundation Trust and University of Bristol<br>Rowe, Edward; North Bristol NHS Trust, Bristol Urology Institute, Department of Urology<br>Koupparis, Anthony; North Bristol NHS Trust, Bristol Urology Institute, Department of Urology<br>Bahl, Amit; University Hospitals Bristol and Weston NHS Foundation Trust, Bristol Haematology and Oncology Centre<br>Shiridzinomwa, Constance; North Bristol NHS Trust, Clinical Research Centre<br>Persad, Raj; North Bristol NHS Trust, Bristol Urology Institute, Department of Urology<br>Martin, Richard; University of Bristol, Bristol Medical School, Population Health Sciences; University of Bristol, National Institute for Health Research Bristol Biomedical Research Centre, University Hospitals Bristol and Weston NHS Foundation Trust and University of Bristol<br>Lane, Athene; University of Bristol, Bristol Medical School, Population Health Sciences; University of Bristol, National Institute for Health Research Bristol Biomedical Research Centre, University Hospitals Bristol and Weston NHS Foundation Trust and University of Bristol

Keywords:	Prostate disease < UROLOGY, Nutritional support < ONCOLOGY, QUALITATIVE RESEARCH

For peer review only - http://bmjopen.bmj.com/site/about/guidelines.xhtml

I, the Submitting Author has the right to grant and does grant on behalf of all authors of the Work (as defined in the below author licence), an exclusive licence and/or a non-exclusive licence for contributions from authors who are: i) UK Crown employees; ii) where BMJ has agreed a CC-BY licence shall apply, and/or iii) in accordance with the terms applicable for US Federal Government officers or employees acting as part of their official duties; on a worldwide, perpetual, irrevocable, royalty-free basis to BMJ Publishing Group Ltd ("BMJ") its licensees and where the relevant Journal is co-owned by BMJ to the co-owners of the Journal, to publish the Work in this journal and any other BMJ products and to exploit all rights, as set out in our licence.

The Submitting Author accepts and understands that any supply made under these terms is made by BMJ to the Submitting Author unless you are acting as an employee on behalf of your employer or a postgraduate student of an affiliated institution which is paying any applicable article publishing charge ("APC") for Open Access articles. Where the Submitting Author wishes to make the Work available on an Open Access basis (and intends to pay the relevant APC), the terms of reuse of such Open Access shall be governed by a Creative Commons licence – details of these licences and which Creative Commons licence will apply to this Work are set out in our licence referred to above.

Other than as permitted in any relevant BMJ Author's Self Archiving Policies, I confirm this Work has not been accepted for publication elsewhere, is not being considered for publication elsewhere and does not duplicate material already published. I confirm all authors consent to publication of this Work and authorise the granting of this licence.

For peer review only

ATTITUDES AND ADHERENCE TO CHANGES IN NUTRITION AND PHYSICAL ACTIVITY FOLLOWING SURGERY FOR PROSTATE CANCER: A QUALITATIVE STUDY

AUTHORS

Corresponding author
Professor J. Athene Lane
Canynge Hall, 39 Whatley Road, Bristol, BS8 2PS
Athene.Lane@bristol.ac.uk

To appear in the order shown below.
* Authors contributed equally.

Dr Luke A Robles
NIHR Bristol Biomedical Research Centre (Nutrition, Diet and Lifestyle theme), University Hospitals Bristol and Weston NHS Foundation Trust and University of Bristol, Bristol, UK

Mrs Ellie Shingler
NIHR Bristol Biomedical Research Centre (Nutrition, Diet and Lifestyle theme), University Hospitals Bristol and Weston NHS Foundation Trust and University of Bristol, Bristol, UK

Dr Lucy McGeagh
Supportive Cancer Care Research Group, Faculty of Health and Life Sciences, Oxford Institute of Nursing, Midwifery and Allied Health Research, Oxford Brookes University, Oxford, UK
NIHR Bristol Biomedical Research Centre (Nutrition, Diet and Lifestyle theme), University Hospitals Bristol and Weston NHS Foundation Trust and University of Bristol, Bristol, UK

Mr Edward Rowe
Bristol Urology Institute, Department of Urology, North Bristol NHS Trust, Bristol, UK

Mr Anthony Koupparis
Bristol Urology Institute, Department of Urology, North Bristol NHS Trust, Bristol, UK

Professor Amit Bahl
Bristol Haematology and Oncology Centre, University Hospitals Bristol and Weston NHS Foundation Trust, Bristol, UK

Mrs Constance Shiridzinomwa
Clinical Research Centre, North Bristol NHS Trust, Bristol, UK

Professor Raj Persad
Bristol Urology Institute, Department of Urology, North Bristol NHS Trust, Bristol, UK

Professor Richard M Martin*
Bristol Medical School, Population Health Sciences, University of Bristol, Bristol, UK
NIHR Bristol Biomedical Research Centre (Nutrition, Diet and Lifestyle theme), University Hospitals Bristol and Weston NHS Foundation Trust and University of Bristol, Bristol, UK

Professor J. Athene Lane*
Bristol Medical School, Population Health Sciences, University of Bristol, Bristol, UK
NIHR Bristol Biomedical Research Centre (Nutrition, Diet and Lifestyle theme), University Hospitals Bristol and Weston NHS Foundation Trust and University of Bristol, Bristol, UK

Word count: 4166

For peer review only - http://bmjopen.bmj.com/site/about/guidelines.xhtml

For peer review only

KEYWORDS

Prostate cancer, adherence, nutrition, physical activity

ABSTRACT

Objectives

Interventions designed to improve men's diet and PA have been recommended as methods of cancer prevention. However, little is known about specific factors that increase men's behavioural intentions and support their adherence, which could inform theory-led interventions. We aimed to explore these factors in men following surgery for prostate cancer.

Design, setting and participants

A qualitative study using semi-structured interviews with men, who made changes to their diet and/or PA as part of a factorial randomised controlled trial conducted at a single hospital in South West England. Participants were 17 men aged 66 years, diagnosed with localised prostate cancer, and underwent surgery. Interview transcripts underwent thematic analysis.

Results

Men were ambivalent about the relationship of nutrition and PA on prostate cancer risk. They believed their diet and level of PA were reasonable before being randomised to their interventions. Men identified several barriers and facilitators to performing these new behaviours. Barriers included nutrition and PA limitations and external obstacles. Facilitators included partner involvement in diet, habit formation, and brisk walking as an individual activity. Men discussed positive effects associated with brisk walking, such as feeling healthier, but not with nutrition interventions. Men in the plant-based diet and brisk walking groups planned to continue these behaviours in the long-term.

Conclusions

The facilitators to behaviour change suggest that adherence to trial interventions can be supported using well-established behaviour change models. Future studies may benefit from theory-based interventions to promote long-term behaviour change in men diagnosed with prostate cancer.

ARTICLE SUMMARY

- This study identified adherence factors relating to men's changes to their diet and PA immediately following surgery.
- The findings are unlikely to represent the experiences of men from other ethnic groups or single men without support with changes to the diet and PA.
- We were unable to provide participant validation of the analysis due to restrictions on time and resources.

BACKGROUND

Prostate cancer is the most common form of cancer in men in the UK with over 48,000 new diagnoses every year [1]. Established risk factors are increasing age, ethnicity (black African or Caribbean), and a family history of prostate cancer [2]. Modifiable factors, such as nutrition and physical activity (PA), have also been linked to prostate cancer risk and progression [3, 4]. A higher intake of cruciferous vegetables (e.g., cabbage,

For peer review only - http://bmjopen.bmj.com/site/about/guidelines.xhtml

For peer review only

cauliflower) is associated with a reduction in prostate cancer incidence and progression [5, 6]. Lycopene, a carotenoid found in many brightly coloured fruit and vegetables, has been linked with reduced risk of cancer progression post-diagnosis [7]. There is also evidence of high intakes of dairy products and calcium is associated with increased prostate cancer risk [8, 9]. With regard to PA, observational studies suggest that moderate to vigorous PA is associated with reduced risk of prostate cancer-specific mortality and biochemical recurrence. More specifically, three hours of moderate to vigorous PA per week is associated with a 61% decrease in prostate cancer mortality compared with less than one hour [10].

The World Cancer Research Fund recommends making changes to nutrition and PA behaviours as methods of cancer prevention [11]. Such behaviour changes include maintaining a plant-based diet (PBD) (i.e. consuming more grains, beans [12], five fruit and vegetables a day [13], and to performing 30 minutes of moderate to vigorous PA a day and limiting sedentary behaviours [14], and the use of supplements, such as lycopene [15]. However, evidence has shown that most cancer survivors do not meet these recommendations. For example, Blanchard and colleagues [16] reported that, out of over 2000 prostate cancer survivors, 43% and 16% were meeting the recommendations of fruit and vegetable consumption and PA respectively.

A systematic review [17] reported that nutrition interventions for cancer populations are rarely guided by behaviour theory. However, theory-based interventions were most effective at improving nutrition changes over a median follow-up of 12 months. Furthermore, constructs of the Theory of Planned Behaviour (TPB) and the Trans-theoretical Model have been shown to increase men's motivation to be more physical active following prostate cancer treatment [18].

Evidence on factors to increase adherence to changes in men's diet and PA is limited. We aimed to identify these factors associated with adherence in men following surgery for localised (organ-confined) prostate cancer.

METHOD

This qualitative study was part of a secondary analysis of a factorial randomised controlled trial (RCT), Prostate cancer Evidence of Exercise and Nutrition Trial (PrEvENT) [19, 20], conducted at a single hospital in South West England. This trial assessed the feasibility and acceptability of nutritional and PA interventions for men after surgery for localised prostate cancer. Details of the trial can be found elsewhere [19]. In brief, men were randomly allocated to nutritional and/or PA interventions (Table 1). This study was written in accordance with the Standards for Reporting Qualitative Research recommendations [21].

Table 1. Nutritional and physical activity interventions

Intervention	Allocation†	Description
Nutritional	Plant-based diet	• 5 fruit and vegetables per day<br>• Substitute dairy milk for non-dairy alternative (e.g., soya, almond or rice milk)
	Lycopene supplementation	• 10mg lycopene capsule taken once per day
	Control	• No changes to usual nutrition
Physical activity	Brisk walking	• 30 minutes brisk walking, 5 times per week
	Control	• No changes to usual daily physical

† Each participant was allocated to both a nutritional and physical activity intervention (factorial randomisation)

For peer review only - http://bmjopen.bmj.com/site/about/guidelines.xhtml

Participants

Seventeen men from the RCT, with an age range of 53 to 81 years (median = 66 years), were recruited into the qualitative element of the study having provided informed consent to be contacted regarding an interview. Purposive sampling was employed to ensure maximum variation across the intervention arms and to ensure that the sample consisted of various demographic characteristics such as age, employment status, and educational level [14]. Trial eligibility included men who were diagnosed with localised prostate cancer, undergoing surgery with no restrictions to performing the interventions. Twenty-five men were approached for interview. Six men were unable to attend due to external circumstances and two men declined giving no reason. Seventeen men agreed and were interviewed. All men interviewed, except one man who was Caribbean, reported themselves as White British or White Other. Most men were retired (n=12) and married (n=13). Over half of the men were educated to secondary school level (n=9) (Table 2).

Table 2. Participant characteristics and intervention allocation

		n = 17	
		n or median	% (range)
Age (years)		66	(53-81)
Ethnicity	White British/White other	16	94
	Caribbean	1	6
Marital status	Married	13	76
	Not married	4	24
Education level	Secondary school	9	53
	University	7	41
	Further education	1	6
Occupation status	Retired	12	71
	Employed	5	29
Intervention arm	Lycopene and brisk walking	4	23
	Lycopene and physical activity control	3	18
	Plant-based diet and brisk walking	3	18
	Plant-based diet and physical activity control	3	18
	Brisk walking and nutritional control	3	18
	Control	1	6

Data collection

Men took part in semi-structured interviews between April 2015 and May 2016 after completing their final 6-month follow-up. Interviews were conducted in-person within a private research clinic room (n=12). For those who were unable to attend in person, a telephone interview was arranged (n=5). Interviews were conducted by three authors (ES, n=9; LM, n=7; LR, n=1). All 3 authors were involved in the data collection process of PrEvENT, although had very minimal contact with participants. Interviews followed a pre-defined interview topic guide (Supplementary material 1). One man in the control group was included in the sampling. They received no intervention aside from standard publicly available nutrition and PA information, if requested. They were asked mainly about their diet and PA before participation in the trial.

Data analysis

Interviews were audio recorded and transcribed for analysis. The transcripts were checked against the audio recordings for accuracy. Data were analysed using an inductive thematic approach with the aid of NVivo 10 software [22]. This involved reading through the transcripts to increase familiarity with the data. They were,

For peer review only - http://bmjopen.bmj.com/site/about/guidelines.xhtml

For peer review only

then, coded for items of data relating to the research question. These codes were collated to form themes, which were reviewed and refined until a coherent narrative of the men's experiences was produced. A constant comparative approach was used to look at differences between sample characteristics, such as age, employment status, and intervention arm. Analysis was conducted in an ongoing manner throughout the data collection process to allow any emergent themes to be further explored in subsequent interviews. This also allowed researchers to identify when data saturation (i.e., no new themes or additional information emerged from the interviews) had been reached.

The coding process was performed by one researcher (ES) and reviewed by a second researcher (LR) to ensure consistency. Emergent themes were reviewed and discussed regularly by both researchers to ensure they remained grounded in the original data. Any inconsistencies found were discussed and resolved through discussion between the researchers and revisiting the transcripts and audio-recordings.

Patient and Public Involvement (PPI)

A prostate cancer PPI group were involved in the concept stages of PrEvENT and reviewed trial documentation, including the interview participant information sheet, consent form, and topic guide.

RESULTS

The analysis yielded five overarching themes: (1) causal beliefs about prostate cancer; (2) perceptions of a healthy diet and PA before diagnosis; (3) barriers to adherence; (4) facilitators of adherence; and (5) perceived benefits of behaviour change.

Causal beliefs about prostate cancer

Men perceived that cancer was caused by external factors such as ageing, genetics, environment agents (i.e., radiation from nuclear sites). When asked about the relationship of diet and PA with cancer, several men believed there was little or no association. Men obtained information about prostate cancer from media sources that were, at times, found to be conflicting.

"…I've looked at these things [causes of cancer] to some extent and I must admit that the evidence for diet-cancer links, to my view, has been weak." P6, PBD and brisk walking

"Well you read it in the paper and sometimes you think there might be [a link with cancer]." P8, lycopene and brisk walking

In contrast, a small number of men reported that they believed that healthy eating and regular exercise was associated with their cancer and this was one reason for maintaining a healthy diet and being physically active.

Interviewer: "…before you took part in the trial, had you ever thought about the links between your lifestyle, what you ate and how much activity you did and cancer?"

Participant: "Well I was concerned that it might be related so I have always tried to eat the right things and do exercise and walking so I just carried on as before. I didn't do any extra walking but I do try and walk at least two miles a day." P13, PBD

For peer review only - http://bmjopen.bmj.com/site/about/guidelines.xhtml

Perceptions of a healthy diet and PA before diagnosis

Men across all the intervention arms believed that they maintained a healthy diet before being diagnosed with prostate cancer. However, the evidence for this notion was mixed. Some men described being able to effectively maintain a healthy diet.

"I really, sort of, eat a fairly Mediterranean diet. I use olive oil instead of butter, for example. If I have a sandwich or something, I put olive oil on. We cook all our own vegetables. I used to have an allotment, which I had to give up because of my leg, because of my knee." P5, lycopene and brisk walking

While other men described making extensive changes on starting the PBD within the trial.

"As I say, I used to eat an awful lot of fruit and vegetables beforehand so I found that I was really, sort of, [toning] myself up almost on fruit and veg. I think it said you had to eat 5 more portions of fruit and veg a day than normal, so I was getting up to, at some stages, about 20, I think, a day." P17, PBD and brisk walking

Men generally described themselves as participating in daily PA, such as going out for regular walks, before being diagnosed. Some men also belonged to a gym.

"I tend to do stretching exercises every day and I do a lot of gardening as well. I love gardening and I do walk. As I say, I've got two little terriers." P15, lycopene

"I'm quite active anyway. Even beforehand I'd get a bike ride, a good two hour or so bike ride once a week and a gym session and once or twice round that walk anyway or sometimes longer. It wasn't a complete change of lifestyle for me." P14, lycopene and brisk walking

Barriers to adherence

Nutritional limitations

Few obstacles were identified by men regarding their adherence to reducing their dairy intake and lycopene supplementations. Some men struggled to adhere to soya milk, mostly due to its taste in coffee or tea. One man believed lycopene caused him some constipation and, therefore, he preferred not to consume his supplements in the long-term.

Interviewer: "If we said, "Can you do this for 12 months?" Could you have carried on?"

Participant: "I would have done yes, but as choice I would say no. I do believe that it causes me slight constipation so I would rather not." P1, lycopene

Physical limitations

Men with co-morbidities, including knee pain, were restricted from walking 'briskly' as this was found to aggravate their physical conditions. Most men also relied on good weather. There was also little motivation to walk elsewhere when the weather was bad.

"…there were some day where it was a total wash-out, and you think, "Well, there's no point in even trying," you know. "I'll make this my quiet day"…" P3, brisk walking and lycopene

For peer review only - http://bmjopen.bmj.com/site/about/guidelines.xhtml

External obstacles

There were also clear differences in men's perceived ability to adhere to brisk walking between those employed and those retired. Work was described as affecting men's success at maintaining their brisk walking.

"Anyway, before I went back to work, it was easy to discipline myself to say, "Right, I'm going to go walking in the morning and in the afternoon, twice a day," but when I went back to work, that wasn't so easy." P6, PBD and brisk walking

"I did think if I'd been working, especially over the winter, it would have been quite difficult to do because you get up and go into work in the dark and come home in the dark." P14, lycopene and brisk walking

Activities that intervened with men's usual routine, such as going on holiday, eating out, and staying with friends were reported to affect some of the men's adherence to both the PBD and brisk walking interventions.

"… I went to my son's [place] and they don't eat a lot of fruit and vegetables there right now, so perhaps for a couple of days then, it was a low count." P2, PBD

"… we were travelling, visiting friends and doing things, so there were some days there when I just couldn't do any walking." P10, brisk walking

Facilitators of adherence

Partner involvement

Men often suggested that their wives or partners would frequently prepare their meals and this would help them with their adherence to the PBD, especially if they also consumed a diet high in fruit and vegetables.

"She is wonderful and she looks after me absolutely, 100%, our food is ready by six…My wife is a three veg, four veg, five veg and, she is greens, she thinks they are wonderful." P2, PBD

Habit formation

Five out of the six men in the lycopene arm were on prescribed medication for other health conditions. They suggested that the routine of self-medicating meant that they found it easy to adhere to taking the supplements.

"It becomes very easy, because the Lycopene, I took every morning with my hypertension medication and it just became part of the breakfast …" P3, lycopene and brisk walking

A couple of brisk walking men, who were physically active and belonged to a gym prior to initiating behaviour changes for the purpose of the trial mentioned that they would overcome barriers, such as bad weather, by incorporating it as part of their usual indoor exercise routine.

"…I built my walk into the gym routine. I did 30 minutes on a treadmill sitting at about 6 kph or something like that with grading…" P14, lycopene and brisk walking

For peer review only - http://bmjopen.bmj.com/site/about/guidelines.xhtml

For peer review only

Brisk walking as individual activity

Although attempts were made by men to carry out their brisk walk with others, most men described that they were happy to walk by themselves and were not dependent on others to help motivate them. Men discussed that one of the reasons why they brisk walked by themselves was due to its intensity (i.e., walking at a pace where they could talk but not sing) as they felt others were not able to walk at the same pace.

"My wife has joined a walking group, but they don't go fast enough. It was too much of an amble. She doesn't walk very fast, by comparison. If ever we're going anywhere, I have to modify my pace to suit her. It was better to do it on my own." P10, brisk walking

Perceived benefits of behaviour change

Most men reported there were many benefits to being more physically active. Several men discussed that going out for a walk provided them with a structured way of performing a reasonable level of PA, which they would not normally do.

"I think, I mean, although I've painted a picture of being quite active, then you know, I mean, it wasn't very organised, you know what I mean? What this did was to impose a routine on me, which I was quite happy with. And it's like setting a goal, isn't it?" P3, lycopene and brisk walking

It also gave them a sense of feeling healthier. One man spoke about how walking to work enabled him to 'clear his head' before starting work. Another man even associated his brisk walking to success of subsequent radiotherapy.

"The walking because it kept me a bit healthier and fitter I think I did better on the radiotherapy." P8, lycopene and brisk walking

Men did not comment on the physical or psychological outcomes they experienced from consuming more fruit and vegetables or lycopene despite knowing the potential health benefits.

DISCUSSION

We aimed to explore factors influencing adherence to nutrition and PA interventions in men, who had surgery following a diagnosis of localised prostate cancer. The findings provided insight into how men perceive a healthy diet and level of PA. They also described men who adhered well to these interventions and indicated factors that both supported and hindered their adherence. Overall, men found the interventions beneficial and those allocated to a PBD and brisk walking interventions planned to continue with their interventions.

The findings suggest that a diagnosis of cancer can provide an opportunity for men to make changes to their nutrition and PA. The trial supports previous evidence that has shown cancer survivors to be highly receptive to changes to their nutrition and PA behaviours. It has been reported that up to 60% of cancer survivors are able to reduce intakes of meat and increased consumption of fruits and vegetables [23]. It has also reported that 49% of prostate cancer survivors can increase their PA post treatment [24].

Men from all the intervention groups believed that they adhered well to their nutrition intervention. While some men followed the intervention guidelines, others made quite extreme changes to their diet, such as eating well-over the recommended daily intake of fruit and vegetables. This suggests that men may benefit from more education on eating practices, including more detail on portion sizes.

For peer review only - http://bmjopen.bmj.com/site/about/guidelines.xhtml

Several factors emerged from the findings that negatively impacted on men's adherence to their interventions. Some men did not like the taste of soya milk and reverted to dairy milk or alternatives forms of diary free milk. This somewhat contradicts findings from previous trials that have shown men to adhere well to a daily consumption of soya products over significant follow-ups. However, these trials incorporated soya products in the form of drink supplements [25, 26] and soya-bread [27]. Thus, the way in which soya products are consumed could influence how men adhere to these products in the long-term. With regard to lycopene, its side-effects are not well-known although other prostate cancer trials have reported diarrhoea and flatulence as plausible side-effects of the supplement in few cases [28]. One man did believe that the constipation he experienced during the trial was due to taking lycopene. Therefore, constipation could potentially be a side-effect and men would need to be aware of these effects and advised how to manage them in future trials.

Barriers to brisk walking included weather conditions, lack of time, and feeling physically constrained due to the weather. These barriers to regular walking have been cited by prostate and other patient populations [29]. Men were assessed for co-morbidities prohibiting them from performing brisk walking before entering PrEvENT. Therefore, it could be speculated that the restrictions to brisk walking reported by men are indicative of their motivation to brisk walk when obstacles arise.

Intention has been cited as an important predictor of men's adherence to exercise interventions [30, 31]. Interventions involving a behavioural component and incorporating techniques, such as problem-solving, have helped to overcome obstacles [32, 33]. PrEvENT used the Theory of Planned Behaviour (TPB) [34] to tailor its patient-facing documents. This theoretical model proposes that behavioural intentions to performing a new health behaviour can be increased through three constructs: (1) having a positive attitude to a behaviour, (2) perceive others to be supportive of it, and (3) believe they can perform the behaviour. RCTs may find it beneficial to use a behaviour change model, such as the TPB, to guide similar interventions. Men identified factors which helped facilitate their adherence to interventions. Partners were found to be significantly involved in choosing and preparing meals for men. Partners are often involved at each stage of men's treatment pathway, including helping them comply with pre-surgery preparation, such as improving fitness and losing weight [35]. Thus, this finding suggests that men would adhere better to PBD interventions with partner involvement.

Men consumed their lycopene along with their usual medication and this existing medication regime supported lycopene adherence. In a similar vein, men who were exercising regularly, before being enrolled in the RCT, were able to include brisk walking into their exercise schedule. Evidence from a previous RCT suggested that men's exercise adherence was more difficult for those who had not considered exercising before entering the trial [30]. Facilitators for lycopene and brisk walking adherence suggest adherence is linked to habitual behaviours (i.e., actions to contextual cues). Habitual formation behaviours have been shown to increase adherence to both nutrition and PA interventions. The current findings suggest incorporating new health behaviours with existing healthy habits could strength adherence [36].

Physical and psychological benefits were reported by those men who brisk walked. These beneficial effects have been reported previously in an observational study measuring PA in cancer survivors, including men with prostate cancer [37]. Those survivors that met the recommended 30 minutes of PA at least 5 times a week had increased health-related quality of life (i.e., physical functioning and better mood states). Such positive outcomes have the potential to help men adhere well to their brisk walking.

Men were not fully convinced that cancer was caused or related to their nutrition or PA. They attributed the cause of their cancer to external factors including age and genetic factors. These findings are partially supported by findings from other qualitative study [38] that included men randomly allocated to dietary interventions. Men believed that cancer was caused by a combination of internal and external factors, including diet being an important factor. However, this sample of men were those at increased risk of

For peer review only - http://bmjopen.bmj.com/site/about/guidelines.xhtml

cancer. Furthermore, a qualitative study [39] has shown that prostate cancer survivors can overestimate the significance of environmental factors, such as pollution and stress, and underestimate behaviour factors associated with increased cancer risk, such as obesity and inactivity. These findings tentatively suggest that men with prostate cancer would adherence to healthy changes to their lifestyle regardless of their perceived association with cancer risk.

Strengths and limitations

A strength of this study is its findings on the psychological, behavioural, and social factors associated with adherence to men's changes to their diet and PA. However, this study has several limitations. All men, except one, were white and the majority were married. It is unlikely that the data fully represents the experiences of men from other ethnic groups or single men without support from partners with their intervention. As this is a qualitative study, findings are based on subjective accounts of behaviour change and there is the chance of men over reporting areas of their behaviour change due to recall bias and men wanted to please the researchers [40]. We were also unable to provide participant validation on the analysis of their individual transcripts due to restrictions on time and resources.

Main implications and future research

The findings suggest that a diagnosis of cancer may influence men's willingness to make changes to their nutrition and PA. This supports the notion of a cancer diagnosis being a 'teachable moment' (events or circumstances that lead to positive behaviour change [41]). Further trials may find approaching men shortly after cancer diagnosis and before commencing treatment to be an opportune time to intervene. Intervention studies should embrace the use of social support to reinforce adherence to dietary changes, especially with PBD interventions where partners are involved with meals prepared at home. Behavioural interventions that can be performed with existing behaviours (e.g., medication regime) are likely to increase participants' confidence and adherence. Further work may want to tailor interventions that consider contextual cues and one's belief in the ability to perform the desired behaviour, as well as behavioural strategies that support adherence. A theory-led behavioural model can both guide and assist with evaluating interventions [42]. The facilitators to change identified in this study (i.e. support from partners, self-efficacy with taking lycopene and exercising) relate to constructs proposed in many behavioural models [43]. Future RCTs could consider utilising behaviour change models to assess and evaluate their support with adherence.

CONCLUSION

The findings from this study may be helpful in developing and implementing future nutrition and PA interventions in men after receiving surgery for prostate cancer. This qualitative study suggests that behaviour change models could help both inform interventions and promote long-term adherence to nutritional and PA behaviours.

ACKNOWLEDGEMENTS

The authors would like to thank all participants for taking part in the qualitative interviews. The authors would also like to thank Dr Aidan Searle for his input on behaviour change theory.

COMPETING INTERESTS

None declared.

For peer review only - http://bmjopen.bmj.com/site/about/guidelines.xhtml

For peer review only

FUNDING

This study was funded by the NIHR Biomedical Research Centre at University Hospitals Bristol and Weston NHS Foundation Trust and the University of Bristol. The views expressed are those of the author(s) and not necessarily those of the NIHR or the Department of Health and Social Care. The funder was not involved in the design of the study and collection, analysis, and interpretation of data and in writing the manuscript.

ETHICS APPROVAL AND CONSENT TO PARTICIPATE

PrEvENT received Research Ethics Committee approval from the National Research Ethics Service Committee South West – Cornwall & Plymouth on 8 April 2014 (REC ref 14/SW/0056). All participants provided written informed consent on enrolment into the trial. Additional oral consent was obtained from participants who took part in telephone interviews. A participant information sheet and consent form were sent in advance of the telephone interviews.

CONSENT FOR PUBLICATION

Not applicable.

DATA AVAILABILITY STATEMENT

The datasets used and/or analysed during the current study is available from the corresponding author on reasonable request.

AUTHOR CONTRIBUTIONS

ES and LHM collected the data. LR, ES, and LHM analysed and interpreted the data and were major contributors to the manuscript. LHM, AB, RP, JAL, and RMM conceived the study, reviewed the article, and critically revised the manuscript. ER, AK, AB, CS, and RP were involved in the study conduct and critically revised the manuscript. All authors read and approved the final manuscript.

REFERENCES

1. Cancer Research UK. Prostate cancer incidence statistics: Cancer Research UK; 2017 [Available from: https://www.cancerresearchuk.org/health-professional/cancer-statistics/statistics-by-cancer-type/prostate-cancer/incidence#heading-Zero.
2. Prostate Cancer UK. Who is at risk? : Prostate Cancer UK; [Available from: https://prostatecanceruk.org/prostate-information/are-you-at-risk#black-men.
3. Stein CJ, Colditz GA. Modifiable risk factors for cancer. Br J Cancer. 2004;90(2):299-303.
4. Peisch SF, Van Blarigan EL, Chan JM, Stampfer MJ, Kenfield SA. Prostate cancer progression and mortality: a review of diet and lifestyle factors. World J Urol. 2016.
5. Richman EL, Carroll PR, Chan JM. Vegetable and fruit intake after diagnosis and risk of prostate cancer progression. International journal of cancer. 2012;131(1):201-10.
6. Liu B, Mao Q, Cao M, Xie L. Cruciferous vegetables intake and risk of prostate cancer: a meta-analysis. International journal of urology : official journal of the Japanese Urological Association. 2012;19(2):134-41.
7. Chan JM, Holick CN, Leitzmann MF, Rimm EB, Willett WC, Stampfer MJ, et al. Diet after diagnosis and the risk of prostate cancer progression, recurrence, and death (United States). Cancer causes & control : CCC. 2006;17(2):199-208.
8. Gao X, LaValley MP, Tucker KL. Prospective studies of dairy product and calcium intakes and prostate cancer risk: a meta-analysis. Journal of the National Cancer Institute. 2005;97(23):1768-77.

For peer review only - http://bmjopen.bmj.com/site/about/guidelines.xhtml

9. Raimondi S, Mabrouk JB, Shatenstein B, Maisonneuve P, Ghadirian P. Diet and prostate cancer risk with specific focus on dairy products and dietary calcium: a case-control study. The Prostate. 2010;70(10):1054-65.
10. Kenfield SA, Stampfer MJ, Giovannucci E, Chan JM. Physical activity and survival after prostate cancer diagnosis in the health professionals follow-up study. Journal of clinical oncology : official journal of the American Society of Clinical Oncology. 2011;29(6):726-32.
11. WCRF/AIRC. Food, nutrition, physical activity and the prevention of cancer: a global perspective Washington, DC: AICR; 2007 [Available from: https://www.wcrf.org/diet-and-cancer/.
12. Parsons JK, Newman VA, Mohler JL, Pierce JP, Flatt S, Marshall J. Dietary modification in patients with prostate cancer on active surveillance: a randomized, multicentre feasibility study. BJU Int. 2008;101(10):1227-31.
13. Demark-Wahnefried W, Peterson B, McBride C, Lipkus I, Clipp E. Current health behaviors and readiness to pursue life-style changes among men and women diagnosed with early stage prostate and breast carcinomas. Cancer. 2000;88(3):674-84.
14. Hackshaw-McGeagh LE, Perry RE, Leach VA, Qandil S, Jeffreys M, Martin RM, et al. A systematic review of dietary, nutritional, and physical activity interventions for the prevention of prostate cancer progression and mortality. Cancer causes & control : CCC. 2015;26(11):1521-50.
15. Bunker CH, McDonald AC, Evans RW, de la Rosa N, Boumosleh JM, Patrick AL. A randomized trial of lycopene supplementation in Tobago men with high prostate cancer risk. Nutrition and cancer. 2007;57(2):130-7.
16. Blanchard CM, Courneya KS, Stein K. Cancer survivors' adherence to lifestyle behavior recommendations and associations with health-related quality of life: results from the American Cancer Society's SCS-II. Journal of clinical oncology : official journal of the American Society of Clinical Oncology. 2008;26(13):2198-204.
17. Avery KN, Donovan JL, Horwood J, Lane JA. Behavior theory for dietary interventions for cancer prevention: a systematic review of utilization and effectiveness in creating behavior change. Cancer causes & control : CCC. 2013;24(3):409-20.
18. Pinto BM, Ciccolo JT. Physical activity motivation and cancer survivorship. Recent results in cancer research Fortschritte der Krebsforschung Progres dans les recherches sur le cancer. 2011;186:367-87.
19. Hackshaw-McGeagh L, Lane JA, Persad R, Gillatt D, Holly JM, Koupparis A, et al. Prostate cancer - evidence of exercise and nutrition trial (PrEvENT): study protocol for a randomised controlled feasibility trial. Trials. 2016;17(1):123.
20. Shingler E, Hackshaw-McGeagh L, Robles L, Persad R, Koupparis A, Rowe E, et al. The feasibility of the Prostate cancer: Evidence of Exercise and Nutrition Trial (PrEvENT) dietary and physical activity modifications: a qualitative study. Trials. 2017;18(1):1-10.
21. O'Brien BC, Harris IB, Beckman TJ, Reed DA, Cook DA. Standards for reporting qualitative research: a synthesis of recommendations. Acad Med. 2014;89(9):1245-51.
22. Ritchie J, Spencer L. Qualitative data analysis for applied policy research. The qualitative researcher's companion. 2002;573:305-29.
23. Demark-Wahnefried W, Aziz NM, Rowland JH, Pinto BM. Riding the crest of the teachable moment: promoting long-term health after the diagnosis of cancer. Journal of clinical oncology : official journal of the American Society of Clinical Oncology. 2005;23(24):5814-30.
24. Thorsen L, Courneya KS, Stevinson C, Fossa SD. A systematic review of physical activity in prostate cancer survivors: outcomes, prevalence, and determinants. Supportive care in cancer : official journal of the Multinational Association of Supportive Care in Cancer. 2008;16(9):987-97.
25. Hamilton-Reeves JM, Rebello SA, Thomas W, Kurzer MS, Slaton JW. Effects of soy protein isolate consumption on prostate cancer biomarkers in men with HGPIN, ASAP, and low-grade prostate cancer. Nutrition and cancer. 2008;60(1):7-13.
26. Bosland MC, Kato I, Zeleniuch-Jacquotte A, Schmoll J, Enk Rueter E, Melamed J, et al. Effect of soy protein isolate supplementation on biochemical recurrence of prostate cancer after radical prostatectomy: a randomized trial. Jama. 2013;310(2):170-8.

For peer review only - http://bmjopen.bmj.com/site/about/guidelines.xhtml

27. Ahn-Jarvis JH, Clinton SK, Grainger EM, Riedl KM, Schwartz SJ, Lee ML, et al. Isoflavone pharmacokinetics and metabolism after consumption of a standardized soy and soy-almond bread in men with asymptomatic prostate cancer. Cancer prevention research (Philadelphia, Pa). 2015;8(11):1045-54.
28. Haseen F, Cantwell MM, O'Sullivan JM, Murray LJ. Is there a benefit from lycopene supplementation in men with prostate cancer? A systematic review. Prostate cancer and prostatic diseases. 2009;12(4):325-32.
29. Rogerson MC, Murphy BM, Bird S, Morris T. "I don't have the heart": a qualitative study of barriers to and facilitators of physical activity for people with coronary heart disease and depressive symptoms. The international journal of behavioral nutrition and physical activity. 2012;9:140.
30. Courneya KS, Segal RJ, Reid RD, Jones LW, Malone SC, Venner PM, et al. Three independent factors predicted adherence in a randomized controlled trial of resistance exercise training among prostate cancer survivors. Journal of Clinical Epidemiology. 2004;57(6):571-9.
31. Speck RM, Courneya KS, Mâsse LC, Duval S, Schmitz KH. An update of controlled physical activity trials in cancer survivors: a systematic review and meta-analysis. Journal of Cancer Survivorship. 2010;4(2):87-100.
32. Bourke L, Doll H, Crank H, Daley A, Rosario D, Saxton JM. Lifestyle intervention in men with advanced prostate cancer receiving androgen suppression therapy: a feasibility study. Cancer epidemiology, biomarkers & prevention : a publication of the American Association for Cancer Research, cosponsored by the American Society of Preventive Oncology. 2011;20(4):647-57.
33. Craike MJ, Livingston PM, Botti M. An exploratory study of the factors that influence physical activity for prostate cancer survivors. Supportive Care in Cancer. 2011;19(7):1019-28.
34. Ajzen I. The theory of planned behavior. Organizational behavior and human decision processes. 1991;50(2):179-211.
35. Kirby R, Holmes K, Amoroso P. SUPPORTING THE SUPPORTER: HELPING THE PARTNER OF PATIENTS NEWLY DIAGNOSED WITH PROSTATE CANCER. BJU International. 2010;105(11):1489-90.
36. Gardner B, Lally P, Wardle J. Making health habitual: the psychology of 'habit-formation'and general practice. Br J Gen Pract. 2012;62(605):664-6.
37. Blanchard CM, Stein KD, Baker F, Dent MF, Denniston MM, Courneya KS, et al. Association between current lifestyle behaviors and health-related quality of life in breast, colorectal, and prostate cancer survivors. Psychology & Health. 2004;19(1):1-13.
38. Horwood J, Avery K, Metcalfe C, Donovan J, Hamdy F, Neal D, et al. Men's knowledge and attitudes towards dietary prevention of a prostate cancer diagnosis: a qualitative study. BMC Cancer. 2014;14(1):812.
39. Wold KS, Byers T, Crane LA, Ahnen D. What do cancer survivors believe causes cancer? (United States). Cancer Causes & Control. 2005;16(2):115-23.
40. Breakwell GM, Hammond S, Fife-Schaw C. Research methods in psychology. Journal of Environmental Psychology. 1994;14(4):335.
41. Lawson PJ, Flocke SA. Teachable moments for health behavior change: a concept analysis. Patient education and counseling. 2009;76(1):25-30.
42. Abraham C, Michie S. A taxonomy of behavior change techniques used in interventions. Health psychology : official journal of the Division of Health Psychology, American Psychological Association. 2008;27(3):379-87.
43. Davis R, Campbell R, Hildon Z, Hobbs L, Michie S. Theories of behaviour and behaviour change across the social and behavioural sciences: a scoping review. Health psychology review. 2015;9(3):323-44.

For peer review only

Patient Interview Topic Guide

PrEvENT: End of trial evaluation interview

Introduction

1. Can you tell me a little about your involvement in the trial?
   a) What arm were you allocated to?
   b) What exactly were you asked to do?

Intervention specifics

2. How would you describe your overall experience of taking part in the intervention?

3. What were the positives of…(intervention arm)

4. Were there any negative elements of…(intervention arm)

5. Would you change any elements of your intervention arm?
   a. Brisk walking for longer / shorter
   b. Add other supplements / take supplements less often
   c. Add more or less changes to diet
      i. If so, which / how?

6. How did you find the instructions you were given about your intervention arm and daily monitoring instructions?
   a. Could you suggest improvements to the instructions?

7. We asked you to make changes to your behaviour approximately 6 weeks after surgery; do you think this was too soon / not soon enough?
   a. What do you think would be the ideal time to make changes to behaviour?

8. How did you find wearing the PA monitoring tool?
   a. Were you able to wear it all of the time? If not, why not?
   b. When were you not able to wear the PA monitoring tool? Why not?
   c. What were barriers to wearing it?
   d. What were benefits to wearing it?
   e. What made it easier?
   f. How did you find the instructions that you were provided with for the monitor?
   g. Could you suggest improvements to the instructions?

9. If we had asked you to stay in the study for longer eg. carrying out the intervention, or completing questionnaires every 6 months, how would you feel about that?
   a. What would make that easier?

For peer review only - http://bmjopen.bmj.com/site/about/guidelines.xhtml

Behaviour change

10. How easy or difficult did you find making changes to your behaviour?
    a. Do you think this would have been different in a different arm? How?

11. Do you think you would be able to continue with these changes for a longer duration of time?

12. How easy or difficult did you find it to remember to carry out the intervention changes?
    a. Why was this?
    b. How did you remember / what advice could you give others to remember?

13. Did you talk to friends / family about the trial and the changes we asked you to make?
    a. How did they respond?
    b. Were they supportive? Critical? Sceptical?
    c. How did that affect you and your behaviours?

14. Have you seen or felt any benefits / negative effects from the changes we asked you to make?

15. Did you see any weight differences due to the change in diet or physical activity?
    a. Would that have made a difference to you?

16. Would you say you have made any lasting changes to your behaviour as a result of the research?
    a. What behavioural changes have you made?
    b. Why did you make these changes?
    c. Why do you plan to continue with these changes?

17. Has participating in the research made you think about your behaviours differently at all?

18. Do you plan to continue with the changes you made?
    a. If so, which? Why? Why not?

19. What are your opinions about the associations between diet, physical activity and cancer?
    a. What about the link with obesity?
    b. Would that make you reconsider your behaviours? What would make you reconsider them?

Trial logistics / contact / nurse appointments

20. How would you describe your experience of the trial form a logistic perspective?
    a. How the appointments were made?
    b. Ease of attending appointments?
    c. Length of appointments?

For peer review only - http://bmjopen.bmj.com/site/about/guidelines.xhtml
For peer review only

21. How did you find your research clinic appointments with the research nurse?
    a. Were these enjoyable?
    b. Did these affect your motivation to continue with the research?
    c. Were you provided with all of the information that you required?

22. How did you find the regular contact / reminders by the research nurse and research team?
    a. Which method did you find the most useful? Why?
    b. Did they affect your motivation?
    c. Do you think they helped you to remember / continue with the trial?

Overall experience

23. How would you sum up your overall experience of taking part in the trial?

24. How would you improve or change the trial to make it better for future participants?

25. Was participating different to what you had expected?
    a. In a positive / negative way?
    b. What had you expected?
    c. Had you participated in research prior to this?

26. Which elements of the trial did you enjoy the most?

27. Which elements of the trial did you not enjoy? Or enjoy the least?

28. Based on your experience in this trial, would you participate in research again?
    a. If so why/if not why not?

29. Final question, what made you agree to take part in the first place?

30. Is there anything further you wish to add?

For peer review only - http://bmjopen.bmj.com/site/about/guidelines.xhtml

Standards for Reporting Qualitative Research (SRQR)

Item no.	Topic/item	Page no.
	**Title and abstract**	
1	**Title** - Concise description of the nature and topic of the study Identifying the study as qualitative or indicating the approach (e.g., ethnography, grounded theory) or data collection methods (e.g., interview, focus group) is recommended	1
2	**Abstract** - Summary of key elements of the study using the abstract format of the intended publication; typically includes background, purpose, methods, results, and conclusions	2
	**Introduction**	
3	**Problem formulation** - Description and significance of the problem/phenomenon studied; review of relevant theory and empirical work; problem statement	3
4	**Purpose or research question** - Purpose of the study and specific objectives or questions	3
	**Methods**	
5	**Qualitative approach and research paradigm** - Qualitative approach (e.g., ethnography, grounded theory, case study, phenomenology, narrative research) and guiding theory if appropriate; identifying the research paradigm (e.g., postpositivist, constructivist/ interpretivist) is also recommended; rationale**	4 - 5
6	**Researcher characteristics and reflexivity** - Researchers' characteristics that may influence the research, including personal attributes, qualifications/experience, relationship with participants, assumptions, and/or presuppositions; potential or actual interaction between researchers' characteristics and the research questions, approach, methods, results, and/or transferability	4
7	**Context** - Setting/site and salient contextual factors; rationale**	4
8	**Sampling strategy** - How and why research participants, documents, or events were selected; criteria for deciding when no further sampling was necessary (e.g., sampling saturation); rationale**	4 - 5
9	**Ethical issues pertaining to human subjects** - Documentation of approval by an appropriate ethics review board and participant consent, or explanation for lack thereof; other confidentiality and data security issues	11
10	**Data collection methods** - Types of data collected; details of data collection procedures including (as appropriate) start and stop dates of data collection and analysis, iterative process, triangulation of sources/methods, and modification of procedures in response to evolving study findings; rationale**	4 - 5
11	**Data collection instruments and technologies** - Description of instruments (e.g., interview guides, questionnaires) and devices (e.g., audio recorders) used for data collection; if/how the instrument(s) changed over the course of the study	4
12	**Units of study** - Number and relevant characteristics of participants, documents, or events included in the study; level of participation (could be reported in results)	Included in results
13	**Data processing** - Methods for processing data prior to and during analysis, including transcription, data entry, data management and security, verification of data integrity, data coding, and anonymization/de-identification of excerpts	4 - 5
14	**Data analysis** - Process by which inferences, themes, etc., were identified and developed, including the researchers involved in data analysis; usually references a specific paradigm or approach; rationale**	4 – 5

For peer review only

15	**Techniques to enhance trustworthiness** - Techniques to enhance trustworthiness and credibility of data analysis (e.g., member checking, audit trail, triangulation); rationale**	10
	**Results/findings**	
16	**Synthesis and interpretation** - Main findings (e.g., interpretations, inferences, and themes); might include development of a theory or model, or integration with prior research or theory	5 – 8
17	**Links to empirical data** - Evidence (e.g., quotes, field notes, text excerpts, photographs) to substantiate analytic findings	5 – 8
	**Discussion**	
18	**Integration with prior work, implications, transferability, and contribution(s) to the field -** Short summary of main findings; explanation of how findings and conclusions connect to, support, elaborate on, or challenge conclusions of earlier scholarship; discussion of scope of application/generalizability; identification of unique contribution(s) to scholarship in a discipline or field	8 – 10
19	**Limitations** - Trustworthiness and limitations of findings	10
	**Other**	
20	**Conflicts of interest** - Potential sources of influence or perceived influence on study conduct and conclusions; how these were managed	10
21	**Funding** - Sources of funding and other support; role of funders in data collection, interpretation, and reporting	11

*The authors created the SRQR by searching the literature to identify guidelines, reporting standards, and critical appraisal criteria for qualitative research; reviewing the reference lists of retrieved sources; and contacting experts to gain feedback. The SRQR aims to improve the transparency of all aspects of qualitative research by providing clear standards for reporting qualitative research.

**The rationale should briefly discuss the justification for choosing that theory, approach, method, or technique rather than other options available, the assumptions and limitations implicit in those choices, and how those choices influence study conclusions and transferability. As appropriate, the rationale for several items might be discussed together.

Citation:

O'Brien, Bridget C. PhD; Harris, Ilene B. PhD; Beckman, Thomas J. MD; Reed, Darcy A. MD, MPH; Cook, David A. MD, MHPE Standards for Reporting Qualitative Research, Academic Medicine: September 2014 - Volume 89 - Issue 9 - p 1245-1251 doi: 10.1097/ACM.0000000000000388.

For peer review only

BMJ Open

ATTITUDES AND ADHERENCE TO CHANGES IN NUTRITION AND PHYSICAL ACTIVITY FOLLOWING SURGERY FOR PROSTATE CANCER: A QUALITATIVE STUDY

Journal:	*BMJ Open*
Manuscript ID	bmjopen-2021-055566.R1
Article Type:	Original research
Date Submitted by the Author:	22-Feb-2022
Complete List of Authors:	Robles, Luke; University of Bristol, National Institute for Health Research Bristol Biomedical Research Centre, University Hospitals Bristol and Weston NHS Foundation Trust and University of Bristol<br>Shingler, Ellie; University of Bristol, National Institute for Health Research Bristol Biomedical Research Centre, University Hospitals Bristol and Weston NHS Foundation Trust and University of Bristol<br>McGeagh, Lucy ; Oxford Brookes University, Supportive Cancer Care Research Group, Faculty of Health and Life Sciences, Oxford Institute of Nursing, Midwifery and Allied Health Research; University of Bristol, National Institute for Health Research Bristol Biomedical Research Centre, University Hospitals Bristol and Weston NHS Foundation Trust and University of Bristol<br>Rowe, Edward; North Bristol NHS Trust, Bristol Urology Institute, Department of Urology<br>Koupparis, Anthony; North Bristol NHS Trust, Bristol Urology Institute, Department of Urology<br>Bahl, Amit; University Hospitals Bristol and Weston NHS Foundation Trust, Bristol Haematology and Oncology Centre<br>Shiridzinomwa, Constance; North Bristol NHS Trust, Clinical Research Centre<br>Persad, Raj; North Bristol NHS Trust, Bristol Urology Institute, Department of Urology<br>Martin, Richard; University of Bristol, Bristol Medical School, Population Health Sciences; University of Bristol, National Institute for Health Research Bristol Biomedical Research Centre, University Hospitals Bristol and Weston NHS Foundation Trust and University of Bristol<br>Lane, Athene; University of Bristol, Bristol Medical School, Population Health Sciences; University of Bristol, National Institute for Health Research Bristol Biomedical Research Centre, University Hospitals Bristol and Weston NHS Foundation Trust and University of Bristol
**Primary Subject Heading**:	Qualitative research
Secondary Subject Heading:	Urology
Keywords:	Prostate disease < UROLOGY, Nutritional support < ONCOLOGY, QUALITATIVE RESEARCH

For peer review only - http://bmjopen.bmj.com/site/about/guidelines.xhtml

SCHOLARONE™
Manuscripts

I, the Submitting Author has the right to grant and does grant on behalf of all authors of the Work (as defined in the below author licence), an exclusive licence and/or a non-exclusive licence for contributions from authors who are: i) UK Crown employees; ii) where BMJ has agreed a CC-BY licence shall apply, and/or iii) in accordance with the terms applicable for US Federal Government officers or employees acting as part of their official duties; on a worldwide, perpetual, irrevocable, royalty-free basis to BMJ Publishing Group Ltd ("BMJ") its licensees and where the relevant Journal is co-owned by BMJ to the co-owners of the Journal, to publish the Work in this journal and any other BMJ products and to exploit all rights, as set out in our licence.

The Submitting Author accepts and understands that any supply made under these terms is made by BMJ to the Submitting Author unless you are acting as an employee on behalf of your employer or a postgraduate student of an affiliated institution which is paying any applicable article publishing charge ("APC") for Open Access articles. Where the Submitting Author wishes to make the Work available on an Open Access basis (and intends to pay the relevant APC), the terms of reuse of such Open Access shall be governed by a Creative Commons licence – details of these licences and which Creative Commons licence will apply to this Work are set out in our licence referred to above.

Other than as permitted in any relevant BMJ Author's Self Archiving Policies, I confirm this Work has not been accepted for publication elsewhere, is not being considered for publication elsewhere and does not duplicate material already published. I confirm all authors consent to publication of this Work and authorise the granting of this licence.

For peer review only

ATTITUDES AND ADHERENCE TO CHANGES IN NUTRITION AND PHYSICAL ACTIVITY FOLLOWING SURGERY FOR PROSTATE CANCER: A QUALITATIVE STUDY

AUTHORS

Corresponding author
Professor J. Athene Lane
Canynge Hall, 39 Whatley Road, Bristol, BS8 2PS
Athene.Lane@bristol.ac.uk

To appear in the order shown below.
* Authors contributed equally.

Dr Luke A Robles
NIHR Bristol Biomedical Research Centre (Nutrition, Diet and Lifestyle theme), University Hospitals Bristol and Weston NHS Foundation Trust and University of Bristol, Bristol, UK

Mrs Ellie Shingler
NIHR Bristol Biomedical Research Centre (Nutrition, Diet and Lifestyle theme), University Hospitals Bristol and Weston NHS Foundation Trust and University of Bristol, Bristol, UK

Dr Lucy McGeagh
Supportive Cancer Care Research Group, Faculty of Health and Life Sciences, Oxford Institute of Nursing, Midwifery and Allied Health Research, Oxford Brookes University, Oxford, UK
NIHR Bristol Biomedical Research Centre (Nutrition, Diet and Lifestyle theme), University Hospitals Bristol and Weston NHS Foundation Trust and University of Bristol, Bristol, UK

Mr Edward Rowe
Bristol Urology Institute, Department of Urology, North Bristol NHS Trust, Bristol, UK

Mr Anthony Koupparis
Bristol Urology Institute, Department of Urology, North Bristol NHS Trust, Bristol, UK

Professor Amit Bahl
Bristol Haematology and Oncology Centre, University Hospitals Bristol and Weston NHS Foundation Trust, Bristol, UK

Mrs Constance Shiridzinomwa
Clinical Research Centre, North Bristol NHS Trust, Bristol, UK

Professor Raj Persad
Bristol Urology Institute, Department of Urology, North Bristol NHS Trust, Bristol, UK

Professor Richard M Martin*
Bristol Medical School, Population Health Sciences, University of Bristol, Bristol, UK
NIHR Bristol Biomedical Research Centre (Nutrition, Diet and Lifestyle theme), University Hospitals Bristol and Weston NHS Foundation Trust and University of Bristol, Bristol, UK

Professor J. Athene Lane*
Bristol Medical School, Population Health Sciences, University of Bristol, Bristol, UK
NIHR Bristol Biomedical Research Centre (Nutrition, Diet and Lifestyle theme), University Hospitals Bristol and Weston NHS Foundation Trust and University of Bristol, Bristol, UK

Word count: 4299

For peer review only - http://bmjopen.bmj.com/site/about/guidelines.xhtml

For peer review only

KEYWORDS

Prostate cancer, adherence, nutrition, physical activity

ABSTRACT

Objectives

Interventions designed to improve men's diet and physical activity (PA) have been recommended as methods of cancer prevention. However, little is known about specific factors that support men's adherence to these health behaviour changes, which could inform theory-led diet and PA interventions. We aimed to explore these factors in men following prostatectomy for prostate cancer (PC).

Design, setting and participants

A qualitative study using semi-structured interviews with men, who made changes to their diet and/or PA as part of a factorial randomised controlled trial conducted at a single hospital in South West England. Participants were 17 men aged 66 years, diagnosed with localised PC, and underwent prostatectomy. Interview transcripts underwent thematic analysis.

Results

Men were ambivalent about the relationship of nutrition and PA on PC risk. They believed their diet and level of PA were reasonable before being randomised to their interventions. Men identified several barriers and facilitators to performing these new behaviours. Barriers included tolerance to dietary changes, PA limitations, and external obstacles. Facilitators included partner involvement in diet, habit formation, and brisk walking as an individual activity. Men discussed positive effects associated with brisk walking, such as feeling healthier, but not with nutrition interventions.

Conclusions

The facilitators to behaviour change suggest that adherence to trial interventions can be supported using well-established behaviour change models. Future studies may benefit from theory-based interventions to support adherence to diet and PA behaviour changes in men diagnosed with PC.

ARTICLE SUMMARY

- This study provided a thematic analysis of men making diet and PA changes soon after prostatectomy, which included a negative case analysis to support the rigour of the study.
- The study included a small sample size of 17 men.
- Data analysis was limited by the lack of depth in men's responses which is likely due to interviews being part of the data collection for a feasibility randomised controlled trial.
- All men, except one, were white indicating that the study sample was not representative of the patient population.

BACKGROUND

Prostate cancer (PC) is the most common form of cancer in men in the UK with over 48,000 new diagnoses every year [1]. Established risk factors are increasing age, ethnicity (black African or Caribbean), and a family history of PC [2]. Modifiable factors, such as nutrition and physical activity (PA), have also been linked to PC risk and progression [3, 4]. A higher intake of cruciferous vegetables (e.g., cabbage, cauliflower) is associated

For peer review only - http://bmjopen.bmj.com/site/about/guidelines.xhtml

with a reduction in PC incidence and progression [5, 6]. Lycopene, a carotenoid found in many brightly coloured fruit and vegetables, has been linked with reduced risk of cancer progression post-diagnosis [7]. High intakes of dairy products is also associated with increased PC risk [8]. With regard to PA, observational studies suggest that moderate to vigorous PA is associated with reduced risk of PC-specific mortality and biochemical recurrence. More specifically, three hours of moderate to vigorous PA per week is associated with a 61% decrease in PC mortality compared with less than one hour [9]. The increase of PA on lower risk of PC-specific mortality and recurrence is supported by intervention studies [10]. In addition, PA has been shown to reduce adverse effects of treatment and improve quality of life, particular in men receiving androgen derivation therapy [11, 12].

The World Cancer Research Fund recommends making changes to nutrition and PA behaviours as methods of cancer prevention [13]. Such behaviour changes include maintaining a plant-based diet (PBD) (i.e. consuming more grains, beans [14], five fruit and vegetables a day [15], and to performing 30 minutes of moderate to vigorous PA a day and limiting sedentary behaviours [16], and the use of supplements, such as lycopene [17]. However, evidence has shown that most cancer survivors do not meet these recommendations. For example, Blanchard and colleagues [18] reported that, out of over 2000 PC survivors, only 43% were meeting the recommendations for fruit and vegetable consumption and only 16% were meeting the recommendations for PA.

A systematic review [19] reported that nutrition interventions for cancer populations are rarely guided by behaviour theory. However, theory-based interventions were most effective at improving nutrition changes over a median follow-up of 12 months. There is limited evidence on psychological and behavioural factors that support adherence to nutrition interventions for men with PC [20]. Furthermore, previous PA intervention studies with patients with chronic conditions, including cancer, have identified several factors that could support adherence to PA [21]. However, few of these studies have explored the psychological and behavioural factors which could align with exist models of behaviour change to enhance PA intervention in men undergoing prostatectomy. For example, a narrative review of behaviour change theories used in PA interventions in urological cancer survivors reported constructs of the Theory of Planned Behaviour (TPB) and the Trans-theoretical Model have been shown to increase men’s motivation to be more physical active either during or following PC treatment [22].

Our qualitative study aimed to identify factors associated with adherence to diet and PA interventions in men following prostatectomy for localised (organ-confined) PC, which could inform such theory-led interventions in this patient population.

METHOD

This descriptive qualitative study was part of a factorial randomised controlled trial (RCT), Prostate cancer Evidence of Exercise and Nutrition Trial (PrEvENT) [23, 24], conducted at a single hospital in South West England. This trial assessed the feasibility and acceptability of nutritional and PA interventions for men after prostatectomy for localised PC. Details of the trial can be found elsewhere (ISRCTN99048944) [23]. In brief, men were randomly allocated to nutritional and/or PA interventions (Table 1). This study was written in accordance with the Standards for Reporting Qualitative Research recommendations [25].

For peer review only - http://bmjopen.bmj.com/site/about/guidelines.xhtml

Table 1. Nutritional and physical activity interventions

Intervention	Allocation†	Description
Nutritional	Plant-based diet	• 5 fruit and vegetables per day<br>• Substitute dairy milk for non-dairy alternative (e.g., soya, almond or rice milk)
	Lycopene supplementation	• 10mg lycopene capsule taken once per day
	Control	• No changes to usual nutrition
Physical activity	Brisk walking	• 30 minutes brisk walking, 5 times per week
	Control	• No changes to usual daily physical

† Each participant was allocated to both a nutritional and physical activity intervention (factorial randomisation)

Participants

Seventeen men from the RCT, with an age range of 53 to 81 years (median = 66 years), were recruited into the qualitative element of the study having provided informed consent to be contacted regarding an interview. Purposive sampling was employed to ensure maximum variation across the intervention arms and to ensure that the sample consisted of various demographic characteristics such as age, employment status, and educational level [14]. Trial eligibility included men who were diagnosed with localised PC, undergoing prostatectomy with no restrictions to performing the interventions. Twenty-five men were approached for interview in person during their 6 months research clinic visit appointment of the feasibility study. Six men were unable to attend due to external and personal circumstances (i.e., did not have the time during the clinic appointment (n=3), interviewer not available (n=2), and participant unwell (n=1)) and two men declined giving no reason. Seventeen men agreed and were interviewed. All men interviewed, except one man who was Caribbean, were reported as White British or White Other. Most men were retired (n=12) and married (n=13). Over half of the men were educated to secondary school level (n=9) (Table 2).

Table 2. Participant characteristics and intervention allocation

		n = 17	
		**n or median**	**% (range)**
Age (years)		66	(53-81)
Ethnicity	White British/White other	16	94
	Caribbean	1	6
Marital status	Married	13	76
	Not married	4	24
Education level	Secondary school (e.g., O-levels, GCSE)	9	53
	University	7	41
	Further education (e.g., A levels, HND)	1	6
Occupation status	Retired	12	71
	Employed	5	29
Intervention arm	Lycopene and brisk walking	4	23
	Lycopene and physical activity control	3	18
	Plant-based diet and brisk walking	3	18
	Plant-based diet and physical activity control	3	18
	Brisk walking and nutritional control	3	18
	Control	1	6

For peer review only - http://bmjopen.bmj.com/site/about/guidelines.xhtml

Data collection

Men took part in semi-structured interviews between April 2015 and May 2016 after completing their final 6-month follow-up. Interviews were conducted in-person within a private research clinic room (n=12). For those who were unable to attend in person, a telephone interview was arranged (n=5). Interviews were conducted by three authors (ES, n=9; LM, n=7; LR, n=1), whose backgrounds include Public Health (ES) and Health Psychology (LM, LR), and lasted between 19 to 84 minutes. All three authors were involved in the data collection process of PrEvENT, although had very minimal contact with participants. Interviews followed a pre-defined interview topic guide (Supplementary material 1), in which questions focused on participants' experiences of performing the interventions from a trial perspective. However, participant responses often included topics associated with long-term adherence to behaviour changes. One man in the control group was included in the sampling. He received no intervention aside from standard publicly available nutrition and PA information, if requested. Data from responses about his diet and PA before participation in the trial was only used for analysis.

Data analysis

Interviews were audio recorded and transcribed for analysis. The transcripts were checked against the audio recordings for accuracy. Data were analysed using inductive thematic analysis with the aid of NVivo 10 software [26]. This method of analysis was chosen with the aim of understanding participant experiences of making behaviour changes beyond those related to study processes of the RCT (e.g., feasibility outcomes). There were also no preconceptions about what themes would be identified from the data. Data analysis involved reading through the transcripts to increase familiarity with the data. They were, then, coded for items of data relating to the research question. The coding process was performed by one researcher (ES) and checked for consistency by a second researcher (LR). These codes were collated to form themes, which were reviewed and refined until a coherent narrative of the men's experiences was produced. Themes were reviewed and discussed regularly by both researchers to ensure they accurately represented the original data. A constant comparative approach was used to look at differences between sample characteristics, such as age, employment status, and intervention arm. Negative case analysis (i.e., identifying contradictory data) were used to broaden or confirm the interpretation of the themes and were resolved through discussion between the researchers and revisiting the transcripts. Data analysis was conducted throughout the data collection process to allow for initial themes to be explored in subsequent interviews. This also allowed researchers to decide when data saturation (i.e., no new themes or additional information emerged from the interviews) was reached.

Patient and Public Involvement (PPI)

A PC PPI group were involved in the concept stages of PrEvENT and reviewed trial documentation, including the interview participant information sheet, consent form, and topic guide.

RESULTS

The analysis yielded five overarching themes: (1) causal beliefs about PC; (2) perceptions of a healthy diet and PA before diagnosis; (3) barriers to adherence; (4) facilitators of adherence; and (5) perceived benefits of behaviour change. The thematic map is shown in Figure 1.

Causal beliefs about PC

Men perceived that cancer was caused by external factors such as ageing, genetics, environment agents (i.e., radiation from nuclear sites). When asked about the impact of diet and PA with cancer, several men

For peer review only - http://bmjopen.bmj.com/site/about/guidelines.xhtml

believed there was little or no association. Men obtained information about PC from media sources that were, at times, found to be conflicting.

"…I've looked at these things [causes of cancer] to some extent and I must admit that the evidence for diet-cancer links, to my view, has been weak." P6, PBD and brisk walking

"Well you read it in the paper and sometimes you think there might be [a link with cancer]." P8, lycopene and brisk walking

In contrast, a small number of men reported that they believed that healthy eating and regular exercise was associated with their cancer and this was one reason for maintaining a healthy diet and being physically active.

Interviewer: "…before you took part in the trial, had you ever thought about the links between your lifestyle, what you ate and how much activity you did and cancer?"

Participant: "Well I was concerned that it might be related so I have always tried to eat the right things and do exercise and walking so I just carried on as before. I didn't do any extra walking but I do try and walk at least two miles a day." P13, PBD

Perceptions of a healthy diet and PA before diagnosis

Men across all the intervention arms believed that they maintained a healthy diet before being diagnosed with PC. However, the evidence for this notion was mixed. Some men described being able to effectively maintain a healthy diet.

"I really, sort of, eat a fairly Mediterranean diet. I use olive oil instead of butter, for example. If I have a sandwich or something, I put olive oil on. We cook all our own vegetables. I used to have an allotment, which I had to give up because of my leg, because of my knee." P5, lycopene and brisk walking

While other men described making extensive changes on starting the PBD within the trial.

"…I found that I was really, sort of, [toning] myself up almost on fruit and veg. I think it said you had to eat 5 more portions of fruit and veg a day than normal, so I was getting up to, at some stages, about 20, I think, a day." P17, PBD and brisk walking

Men generally described themselves as participating in daily PA, such as going out for regular walks, before being diagnosed. Some men also belonged to a gym.

"I tend to do stretching exercises every day and I do a lot of gardening as well. I love gardening and I do walk. As I say, I've got two little terriers." P15, lycopene

"I'm quite active anyway. Even beforehand I'd get a bike ride, a good two hour or so bike ride once a week and a gym session and once or twice round that walk anyway or sometimes longer. It wasn't a complete change of lifestyle for me." P14, lycopene and brisk walking

For peer review only - http://bmjopen.bmj.com/site/about/guidelines.xhtml

Barriers to adherence

Tolerance of the interventions

Few obstacles were identified by men regarding their ability to adherence to the interventions. Men with co-morbidities, including knee pain, were restricted from walking 'briskly' as this was found to aggravate their physical conditions. Some men struggled to adhere to soya milk, mostly due to its taste in coffee or tea. One man believed lycopene caused him some constipation and, therefore, he preferred not to consume his supplements in the long-term.

Interviewer: "If we said, "Can you do this for 12 months?" Could you have carried on?"

Participant: "I would have done yes, but as choice I would say no. I do believe that it causes me slight constipation so I would rather not." P10, lycopene

External obstacles

Most men relied on good weather. There was also little motivation to walk elsewhere when the weather was bad.

"…there were some day where it was a total wash-out, and you think, "Well, there's no point in even trying," you know. "I'll make this my quiet day"…" P3, brisk walking and lycopene

There were also clear differences in men's perceived ability to adhere to brisk walking between those employed and those retired. Work was described as affecting men's success at maintaining their brisk walking.

"Anyway, before I went back to work, it was easy to discipline myself to say, "Right, I'm going to go walking in the morning and in the afternoon, twice a day," but when I went back to work, that wasn't so easy." P6, PBD and brisk walking

"I did think if I'd been working, especially over the winter, it would have been quite difficult to do because you get up and go into work in the dark and come home in the dark." P14, lycopene and brisk walking

Activities that intervened with men's usual routine, such as going on holiday, eating out, and staying with friends were reported to affect some of the men's adherence to both the PBD and brisk walking interventions.

"… I went to my son's [place] and they don't eat a lot of fruit and vegetables there right now, so perhaps for a couple of days then, it was a low count." P2, PBD

"… we were travelling, visiting friends and doing things, so there were some days there when I just couldn't do any walking." P1, brisk walking

Facilitators of adherence

Partner involvement

Men often suggested that their wives or partners would frequently prepare their meals and this would help them with their adherence to the PBD, especially if they also consumed a diet high in fruit and vegetables.

For peer review only - http://bmjopen.bmj.com/site/about/guidelines.xhtml

"She is wonderful and she looks after me absolutely, 100%, our food is ready by six…My wife is a three veg, four veg, five veg and, she is greens, she thinks they are wonderful." P2, PBD

Habit formation

Five out of the six men in the lycopene arm were on prescribed medication for other health conditions. They suggested that the routine of self-medicating meant that they found it easy to adhere to taking the supplements.

"It becomes very easy, because the Lycopene, I took every morning with my hypertension medication and it just became part of the breakfast …" P3, lycopene and brisk walking

A couple of brisk walking men, who were physically active and belonged to a gym prior to initiating behaviour changes for the purpose of the trial mentioned that they would overcome barriers, such as bad weather, by incorporating it as part of their usual indoor exercise routine.

"…I built my walk into the gym routine. I did 30 minutes on a treadmill sitting at about 6 kph or something like that with grading…" P14, lycopene and brisk walking

Brisk walking as individual activity

Although attempts were made by men to carry out their brisk walk with others, most men claimed that they were happy to walk by themselves and were not dependent on others to help motivate them. Men discussed that one of the reasons why they brisk walked by themselves was due to its intensity (i.e., walking at a pace where they could talk but not sing) as they felt others were not able to walk at the same pace.

"My wife has joined a walking group, but they don't go fast enough. It was too much of an amble. She doesn't walk very fast, by comparison. If ever we're going anywhere, I have to modify my pace to suit her. It was better to do it on my own." P1, brisk walking

Perceived benefits of behaviour change

Most men reported there were many benefits to being more physically active. Several men discussed that going out for a walk provided them with a structured way of performing a reasonable level of PA, which they would not normally do.

"I think, I mean, although I've painted a picture of being quite active, then you know, I mean, it wasn't very organised, you know what I mean? What this did was to impose a routine on me, which I was quite happy with. And it's like setting a goal, isn't it?" P3, lycopene and brisk walking

It also gave them a sense of feeling healthier. One man spoke about how walking to work enabled him to 'clear his head' before starting work. Another man even associated his brisk walking to success of subsequent radiotherapy.

"The walking because it kept me a bit healthier and fitter I think I did better on the radiotherapy." P8, lycopene and brisk walking

Men did not comment on the physical or psychological outcomes they experienced from consuming more fruit and vegetables or lycopene despite knowing the potential health benefits.

For peer review only - http://bmjopen.bmj.com/site/about/guidelines.xhtml

DISCUSSION

Summary of findings

We aimed to explore factors influencing adherence to nutrition and PA interventions in men, who had prostatectomy following a diagnosis of localised PC. Our findings showed that men believed their cancer was caused by external factors, such as age and genetics. They discussed eating healthily and regularly exercising before their diagnosis and barriers and facilitators to their behaviour changes. Overall, men found the PA intervention beneficial to their health and wellbeing.

Support with other studies

Men were not fully convinced that cancer was caused or related to their nutrition or PA. They attributed the cause of their cancer to external factors including age and genetic factors. These findings are supported by another qualitative study [27], which shown that PC survivors can overestimate the significance of environmental factors, such as pollution and stress, and underestimate behaviour factors associated with increased cancer risk, such as obesity and inactivity. In contrast, observational evidence showed that a high proportion of women attributed diet (68%), to their breast cancer diagnosis in addition to external factors (i.e. hormones) [28]. These findings could be indicative of men's preference to believe in causal factors that are outside their control.

Men from all the intervention groups believed that they adhered well to their nutrition intervention. While some men followed the intervention guidelines, others made quite extreme changes to their diet, such as eating well-over the recommended daily intake of fruit and vegetables. This suggests that men may benefit from more education on eating practices, including more detail on portion sizes.

Men's tolerance to changes in their diet impacted on their adherence to their nutrition interventions. Some men did not like the taste of soya milk and reverted to dairy milk or alternatives forms of diary free milk. This somewhat contradicts findings from previous trials that have shown men to adhere well to a daily consumption of soya products over significant follow-ups. However, these trials incorporated soya products in the form of drink supplements [29, 30] and soya-bread [31]. Thus, the way in which soya products are consumed could influence how men adhere to these products in the long-term. With regard to lycopene, its side-effects are not well-known although other PC trials have reported diarrhoea and flatulence as plausible side-effects of the supplement in few cases [32]. One man did believe that the constipation he experienced during the trial was due to taking lycopene. Therefore, constipation could potentially be a side-effect and men would need to be aware of these effects and advised how to manage them in future trials.

Barriers to brisk walking included weather conditions and a lack of time. These barriers to regular walking have been cited by prostate and other patient populations [33] [34]. Men were assessed for co-morbidities prohibiting them from performing brisk walking before entering PrEvENT. Therefore, it could be speculated that the physical restrictions to brisk walking reported by men are indicative of their motivation to brisk walk when obstacles arise.

Partners were found to be significantly involved in choosing and preparing meals for men. Partners are often involved at each stage of men's treatment pathway, including helping them comply with pre-prostatectomy preparation, such as improving fitness and losing weight [35]. Thus, this finding suggests that men would adhere better to PBD interventions with partner involvement. In contrast, men discussed the PA intervention as one which they preferred to do by themselves. This finding somewhat contradicts evidence which has shown men to report physical, mental and relationship benefits from PA interventions involving their partners [36]. However, the prescribed nature of couple-based interventions is likely to attribute to its efficacy.

For peer review only - http://bmjopen.bmj.com/site/about/guidelines.xhtml

A facilitator to the lycopene intervention was men consuming the supplement along with their existing medication regime. In a similar vein, men who were exercising regularly, before being enrolled in the RCT, were able to include brisk walking into their exercise schedule. Evidence from a previous RCT suggested that men's exercise adherence was more difficult for those who had not considered exercising before entering the trial [37]. These facilitators for lycopene and brisk walking adherence suggest adherence is linked to habitual behaviours (i.e., actions to contextual cues). Habitual formation behaviours have been shown to increase adherence to both nutrition and PA interventions. The current findings suggest incorporating new health behaviours with existing healthy habits could strength adherence [38].

Physical and psychological benefits were reported by those men who brisk walked. These beneficial effects have been reported in a prospective study measuring PA in men with PC [39]. Men who adhered to 150 minutes of moderate PA post-diagnosis had significantly better physical ($\beta = 6.01$, 95% confidence interval (CI): 4.15 to 7.86) and mental ($\beta = 2.32$; 95% CI: 0.29-4.34) quality of life (i.e., physical functioning and better mood states) compared to men who were non-adherent. Such positive outcomes have the potential to help men adhere well to their brisk walking.

Strengths and limitations

The strength of this study is that it has provided a thematic analysis of men making diet and PA changes soon after prostatectomy, which included a negative case analysis to support the rigour of the study. However, this study has several limitations. Data analysis was limited by the lack of depth in responses from the interviews. This is likely due to the interviews being part of data collection for a feasibility RCT, which assessed trial processes as well as intervention adherence. Therefore, further assessments of rigour would not have benefitted the data analysis. The sample size was small (n=17) and all men, except one, were white and the majority were married. It is unlikely that the data fully represents the experiences of men from other ethnic groups or single men without support from partners with their intervention. In addition, men in all the intervention arms discussed that they were already maintaining a healthy diet and engaging in regular physical activities before their diagnosis. This could suggest that the current findings are limited to men more willing and able to perform these health behaviours. As this is a qualitative study, findings are based on subjective accounts of behaviour change and there is the chance of men over reporting areas of their behaviour change due to recall bias and men wanted to please the researchers [40].

Main implications and future research

Intervention studies should embrace the use of social support to reinforce adherence to dietary changes, especially with PBD interventions where partners are involved with meals prepared at home. Behavioural interventions that can be performed with existing behaviours (e.g., medication regime) are likely to increase participants' confidence and adherence. Further work may want to tailor interventions that consider contextual cues and one's belief in the ability to perform the desired behaviour, as well as behavioural strategies that support adherence. A theory-led behavioural model can both guide and assist with evaluating interventions [41]. Our study findings indicate that men are motivated to make changes to their diet and level of PA following prostatectomy. However, men's motivation was not related to beliefs that diet and PA was associated with their PC. Other psychological factors could explain men's motivation to adherence to these behaviour changes, such as symptom control, which could be explored using qualitative studies. Barriers to adhering to their behaviour changes related to physical (i.e., weather, time) and social opportunities (e.g., going on holiday). These findings suggest that future nutrition and PA interventions guided by a behavioural model, which help identify these barriers and incorporate techniques such as problem-solving, will improve adherence [42, 43]. The COM-B model [44] could be one that is suitable for this patient population. This model proposes that a person's motivation to perform and maintain a behaviour is supported by their capability (i.e., psychologically and physically) and opportunity (i.e., social

For peer review only - http://bmjopen.bmj.com/site/about/guidelines.xhtml

and physical) to perform the behaviour. Future studies may consider exploring the use of this model in nutrition and PA intervention studies with PC populations.

CONCLUSION

The findings from this study may be helpful in developing and implementing future nutrition and PA interventions in men after receiving prostatectomy for PC. This qualitative study suggests that behaviour change models could support adherence to nutritional and PA behaviours.

ACKNOWLEDGEMENTS

The authors would like to thank all participants for taking part in the qualitative interviews. The authors would also like to thank Dr Aidan Searle for his input on behaviour change theory.

COMPETING INTERESTS

None declared.

FUNDING

This study was funded by the NIHR Biomedical Research Centre at University Hospitals Bristol and Weston NHS Foundation Trust and the University of Bristol. The views expressed are those of the author(s) and not necessarily those of the NIHR or the Department of Health and Social Care. The funder was not involved in the design of the study and collection, analysis, and interpretation of data and in writing the manuscript.

ETHICS APPROVAL AND CONSENT TO PARTICIPATE

PrEvENT received Research Ethics Committee approval from the National Research Ethics Service Committee South West – Cornwall & Plymouth on 8 April 2014 (REC ref 14/SW/0056). All participants provided written informed consent on enrolment into the trial. Additional oral consent was obtained from participants who took part in telephone interviews. A participant information sheet and consent form were sent in advance of the telephone interviews.

CONSENT FOR PUBLICATION

Not applicable.

DATA AVAILABILITY STATEMENT

The datasets used and/or analysed during the current study is available from the corresponding author on reasonable request.

AUTHOR CONTRIBUTIONS

ES and LHM collected the data. LR, ES, and LHM analysed and interpreted the data and were major contributors to the manuscript. LHM, AB, RP, JAL, and RMM conceived the study, reviewed the article, and critically revised the manuscript. ER, AK, AB, CS, and RP were involved in the study conduct and critically revised the manuscript. All authors read and approved the final manuscript.

For peer review only

For peer review only - http://bmjopen.bmj.com/site/about/guidelines.xhtml

REFERENCES

1. Cancer Research UK. Prostate cancer incidence statistics: Cancer Research UK; 2017 [Available from: https://www.cancerresearchuk.org/health-professional/cancer-statistics/statistics-by-cancer-type/prostate-cancer/incidence#heading-Zero.
2. Prostate Cancer UK. Who is at risk? : Prostate Cancer UK; [Available from: https://prostatecanceruk.org/prostate-information/are-you-at-risk#black-men.
3. Stein CJ, Colditz GA. Modifiable risk factors for cancer. British journal of cancer. 2004;90(2):299-303.
4. Peisch SF, Van Blarigan EL, Chan JM, Stampfer MJ, Kenfield SA. Prostate cancer progression and mortality: a review of diet and lifestyle factors. World journal of urology. 2016.
5. Richman EL, Carroll PR, Chan JM. Vegetable and fruit intake after diagnosis and risk of prostate cancer progression. International journal of cancer. 2012;131(1):201-10.
6. Liu B, Mao Q, Cao M, Xie L. Cruciferous vegetables intake and risk of prostate cancer: a meta-analysis. International journal of urology : official journal of the Japanese Urological Association. 2012;19(2):134-41.
7. Chan JM, Holick CN, Leitzmann MF, Rimm EB, Willett WC, Stampfer MJ, et al. Diet after diagnosis and the risk of prostate cancer progression, recurrence, and death (United States). Cancer causes & control : CCC. 2006;17(2):199-208.
8. López-Plaza B, Bermejo LM, Santurino C, Cavero-Redondo I, Álvarez-Bueno C, Gómez-Candela C. Milk and Dairy Product Consumption and Prostate Cancer Risk and Mortality: An Overview of Systematic Reviews and Meta-analyses. Advances in Nutrition. 2019;10(suppl_2):S212-S23.
9. Kenfield SA, Stampfer MJ, Giovannucci E, Chan JM. Physical activity and survival after prostate cancer diagnosis in the health professionals follow-up study. Journal of clinical oncology : official journal of the American Society of Clinical Oncology. 2011;29(6):726-32.
10. Cormie P, Zopf EM, Zhang X, Schmitz KH. The Impact of Exercise on Cancer Mortality, Recurrence, and Treatment-Related Adverse Effects. Epidemiologic Reviews. 2017;39(1):71-92.
11. Bressi B, Cagliari M, Contesini M, Mazzini E, Bergamaschi FAM, Moscato A, et al. Physical exercise for bone health in men with prostate cancer receiving androgen deprivation therapy: a systematic review. Support Care Cancer. 2021;29(4):1811-24.
12. Cagliari M, Bressi B, Bassi MC, Fugazzaro S, Prati G, Iotti C, et al. Feasibility and Safety of Physical Exercise to Preserve Bone Health in Men with Prostate Cancer Receiving Androgen Deprivation Therapy: A Systematic Review. Phys Ther. 2021.
13. WCRF/AIRC. Food, nutrition, physical activity and the prevention of cancer: a global perspective Washington, DC: AICR; 2007 [Available from: https://www.wcrf.org/diet-and-cancer/.
14. Parsons JK, Newman VA, Mohler JL, Pierce JP, Flatt S, Marshall J. Dietary modification in patients with prostate cancer on active surveillance: a randomized, multicentre feasibility study. BJU international. 2008;101(10):1227-31.
15. Demark-Wahnefried W, Peterson B, McBride C, Lipkus I, Clipp E. Current health behaviors and readiness to pursue life-style changes among men and women diagnosed with early stage prostate and breast carcinomas. Cancer. 2000;88(3):674-84.
16. Hackshaw-McGeagh LE, Perry RE, Leach VA, Qandil S, Jeffreys M, Martin RM, et al. A systematic review of dietary, nutritional, and physical activity interventions for the prevention of prostate cancer progression and mortality. Cancer causes & control : CCC. 2015;26(11):1521-50.
17. Bunker CH, McDonald AC, Evans RW, de la Rosa N, Boumosleh JM, Patrick AL. A randomized trial of lycopene supplementation in Tobago men with high prostate cancer risk. Nutrition and cancer. 2007;57(2):130-7.
18. Blanchard CM, Courneya KS, Stein K. Cancer survivors' adherence to lifestyle behavior recommendations and associations with health-related quality of life: results from the American Cancer Society's SCS-II. Journal of clinical oncology : official journal of the American Society of Clinical Oncology. 2008;26(13):2198-204.

For peer review only - http://bmjopen.bmj.com/site/about/guidelines.xhtml

19. Avery KN, Donovan JL, Horwood J, Lane JA. Behavior theory for dietary interventions for cancer prevention: a systematic review of utilization and effectiveness in creating behavior change. Cancer causes & control : CCC. 2013;24(3):409-20.
20. Stacey FG, James EL, Chapman K, Courneya KS, Lubans DR. A systematic review and meta-analysis of social cognitive theory-based physical activity and/or nutrition behavior change interventions for cancer survivors. Journal of Cancer Survivorship. 2015;9(2):305-38.
21. Collado-Mateo D, Lavín-Pérez AM, Peñacoba C, Del Coso J, Leyton-Román M, Luque-Casado A, et al. Key Factors Associated with Adherence to Physical Exercise in Patients with Chronic Diseases and Older Adults: An Umbrella Review. International Journal of Environmental Research and Public Health. 2021;18(4):2023.
22. Rammant E, Bultijnck R, Caperchione CM, Trinh L. The Use of Theory to Develop Physical Activity Interventions in Urological Cancer Survivors: A Narrative Review. Seminars in Oncology Nursing. 2021;37(1):151109.
23. Hackshaw-McGeagh L, Lane JA, Persad R, Gillatt D, Holly JM, Koupparis A, et al. Prostate cancer - evidence of exercise and nutrition trial (PrEvENT): study protocol for a randomised controlled feasibility trial. Trials. 2016;17(1):123.
24. Shingler E, Hackshaw-McGeagh L, Robles L, Persad R, Koupparis A, Rowe E, et al. The feasibility of the Prostate cancer: Evidence of Exercise and Nutrition Trial (PrEvENT) dietary and physical activity modifications: a qualitative study. Trials. 2017;18(1):1-10.
25. O'Brien BC, Harris IB, Beckman TJ, Reed DA, Cook DA. Standards for reporting qualitative research: a synthesis of recommendations. Acad Med. 2014;89(9):1245-51.
26. Ritchie J, Spencer L. Qualitative data analysis for applied policy research. The qualitative researcher's companion. 2002;573:305-29.
27. Wold KS, Byers T, Crane LA, Ahnen D. What do cancer survivors believe causes cancer? (United States). Cancer Causes & Control. 2005;16(2):115-23.
28. Costanzo ES, Lutgendorf SK, Roeder SL. Common-sense beliefs about cancer and health practices among women completing treatment for breast cancer. Psychooncology. 2011;20(1):53-61.
29. Hamilton-Reeves JM, Rebello SA, Thomas W, Kurzer MS, Slaton JW. Effects of soy protein isolate consumption on prostate cancer biomarkers in men with HGPIN, ASAP, and low-grade prostate cancer. Nutrition and cancer. 2008;60(1):7-13.
30. Bosland MC, Kato I, Zeleniuch-Jacquotte A, Schmoll J, Enk Rueter E, Melamed J, et al. Effect of soy protein isolate supplementation on biochemical recurrence of prostate cancer after radical prostatectomy: a randomized trial. Jama. 2013;310(2):170-8.
31. Ahn-Jarvis JH, Clinton SK, Grainger EM, Riedl KM, Schwartz SJ, Lee ML, et al. Isoflavone pharmacokinetics and metabolism after consumption of a standardized soy and soy-almond bread in men with asymptomatic prostate cancer. Cancer prevention research (Philadelphia, Pa). 2015;8(11):1045-54.
32. Haseen F, Cantwell MM, O'Sullivan JM, Murray LJ. Is there a benefit from lycopene supplementation in men with prostate cancer? A systematic review. Prostate cancer and prostatic diseases. 2009;12(4):325-32.
33. Hackshaw-McGeagh LE, Sutton E, Persad R, Aning J, Bahl A, Koupparis A, et al. Acceptability of dietary and physical activity lifestyle modification for men following radiotherapy or radical prostatectomy for localised prostate cancer: a qualitative investigation. BMC Urology. 2017;17(1):94.
34. Rogerson MC, Murphy BM, Bird S, Morris T. "I don't have the heart": a qualitative study of barriers to and facilitators of physical activity for people with coronary heart disease and depressive symptoms. The international journal of behavioral nutrition and physical activity. 2012;9:140.
35. Kirby R, Holmes K, Amoroso P. SUPPORTING THE SUPPORTER: HELPING THE PARTNER OF PATIENTS NEWLY DIAGNOSED WITH PROSTATE CANCER. BJU international. 2010;105(11):1489-90.
36. Winters-Stone KM, Lyons KS, Beer TM, Skiba MB, Hung A. A pilot feasibility study of Exercising Together© during radiation therapy for prostate cancer: a dyadic approach for patients and spouses. Pilot and Feasibility Studies. 2021;7(1):216.

For peer review only - http://bmjopen.bmj.com/site/about/guidelines.xhtml

37. Courneya KS, Segal RJ, Reid RD, Jones LW, Malone SC, Venner PM, et al. Three independent factors predicted adherence in a randomized controlled trial of resistance exercise training among prostate cancer survivors. Journal of Clinical Epidemiology. 2004;57(6):571-9.

38. Gardner B, Lally P, Wardle J. Making health habitual: the psychology of 'habit-formation'and general practice. Br J Gen Pract. 2012;62(605):664-6.

39. Farris MS, Kopciuk KA, Courneya KS, McGregor SE, Wang Q, Friedenreich CM. Associations of Postdiagnosis Physical Activity and Change from Prediagnosis Physical Activity with Quality of Life in Prostate Cancer Survivors. Cancer epidemiology, biomarkers & prevention : a publication of the American Association for Cancer Research, cosponsored by the American Society of Preventive Oncology. 2017;26(2):179-87.

40. Breakwell GM, Hammond S, Fife-Schaw C. Research methods in psychology. Journal of Environmental Psychology. 1994;14(4):335.

41. Abraham C, Michie S. A taxonomy of behavior change techniques used in interventions. Health psychology : official journal of the Division of Health Psychology, American Psychological Association. 2008;27(3):379-87.

42. Bourke L, Doll H, Crank H, Daley A, Rosario D, Saxton JM. Lifestyle intervention in men with advanced prostate cancer receiving androgen suppression therapy: a feasibility study. Cancer epidemiology, biomarkers & prevention : a publication of the American Association for Cancer Research, cosponsored by the American Society of Preventive Oncology. 2011;20(4):647-57.

43. Craike MJ, Livingston PM, Botti M. An exploratory study of the factors that influence physical activity for prostate cancer survivors. Supportive Care in Cancer. 2011;19(7):1019-28.

44. Michie S, van Stralen MM, West R. The behaviour change wheel: a new method for characterising and designing behaviour change interventions. Implement Sci. 2011;6:42.

LEGEND

Figure 1: Thematic map of qualitative analysis (PA, physical activity)

For peer review only

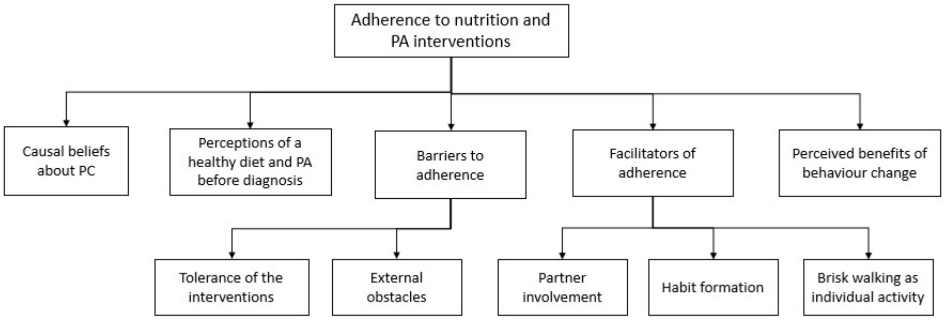

Figure 1: Thematic map of qualitative analysis (PA, physical activity)

247x104mm (96 x 96 DPI)

Patient Interview Topic Guide

PrEvENT: End of trial evaluation interview

<u>Introduction</u>

1. Can you tell me a little about your involvement in the trial?
   a) What arm were you allocated to?
   b) What exactly were you asked to do?

<u>Intervention specifics</u>

2. How would you describe your overall experience of taking part in the intervention?

3. What were the positives of…(intervention arm)

4. Were there any negative elements of…(intervention arm)

5. Would you change any elements of your intervention arm?
   a. Brisk walking for longer / shorter
   b. Add other supplements / take supplements less often
   c. Add more or less changes to diet
      i. If so, which / how?

6. How did you find the instructions you were given about your intervention arm and daily monitoring instructions?
   a. Could you suggest improvements to the instructions?

7. We asked you to make changes to your behaviour approximately 6 weeks after surgery; do you think this was too soon / not soon enough?
   a. What do you think would be the ideal time to make changes to behaviour?

8. How did you find wearing the PA monitoring tool?
   a. Were you able to wear it all of the time? If not, why not?
   b. When were you not able to wear the PA monitoring tool? Why not?
   c. What were barriers to wearing it?
   d. What were benefits to wearing it?
   e. What made it easier?
   f. How did you find the instructions that you were provided with for the monitor?
   g. Could you suggest improvements to the instructions?

9. If we had asked you to stay in the study for longer eg. carrying out the intervention, or completing questionnaires every 6 months, how would you feel about that?
   a. What would make that easier?

For peer review only

For peer review only - http://bmjopen.bmj.com/site/about/guidelines.xhtml

Behaviour change

10. How easy or difficult did you find making changes to your behaviour?
    a. Do you think this would have been different in a different arm? How?

11. Do you think you would be able to continue with these changes for a longer duration of time?

12. How easy or difficult did you find it to remember to carry out the intervention changes?
    a. Why was this?
    b. How did you remember / what advice could you give others to remember?

13. Did you talk to friends / family about the trial and the changes we asked you to make?
    a. How did they respond?
    b. Were they supportive? Critical? Sceptical?
    c. How did that affect you and your behaviours?

14. Have you seen or felt any benefits / negative effects from the changes we asked you to make?

15. Did you see any weight differences due to the change in diet or physical activity?
    a. Would that have made a difference to you?

16. Would you say you have made any lasting changes to your behaviour as a result of the research?
    a. What behavioural changes have you made?
    b. Why did you make these changes?
    c. Why do you plan to continue with these changes?

17. Has participating in the research made you think about your behaviours differently at all?

18. Do you plan to continue with the changes you made?
    a. If so, which? Why? Why not?

19. What are your opinions about the associations between diet, physical activity and cancer?
    a. What about the link with obesity?
    b. Would that make you reconsider your behaviours? What would make you reconsider them?

Trial logistics / contact / nurse appointments

20. How would you describe your experience of the trial form a logistic perspective?
    a. How the appointments were made?
    b. Ease of attending appointments?
    c. Length of appointments?

For peer review only

For peer review only - http://bmjopen.bmj.com/site/about/guidelines.xhtml

21. How did you find your research clinic appointments with the research nurse?
    a. Were these enjoyable?
    b. Did these affect your motivation to continue with the research?
    c. Were you provided with all of the information that you required?

22. How did you find the regular contact / reminders by the research nurse and research team?
    a. Which method did you find the most useful? Why?
    b. Did they affect your motivation?
    c. Do you think they helped you to remember / continue with the trial?

Overall experience

23. How would you sum up your overall experience of taking part in the trial?

24. How would you improve or change the trial to make it better for future participants?

25. Was participating different to what you had expected?
    a. In a positive / negative way?
    b. What had you expected?
    c. Had you participated in research prior to this?

26. Which elements of the trial did you enjoy the most?

27. Which elements of the trial did you not enjoy? Or enjoy the least?

28. Based on your experience in this trial, would you participate in research again?
    a. If so why/if not why not?

29. Final question, what made you agree to take part in the first place?

30. Is there anything further you wish to add?

For peer review only

For peer review only - http://bmjopen.bmj.com/site/about/guidelines.xhtml

Standards for Reporting Qualitative Research (SRQR)

Item no.	Topic/item	Page no.
	**Title and abstract**	
1	**Title** - Concise description of the nature and topic of the study Identifying the study as qualitative or indicating the approach (e.g., ethnography, grounded theory) or data collection methods (e.g., interview, focus group) is recommended	1
2	**Abstract** - Summary of key elements of the study using the abstract format of the intended publication; typically includes background, purpose, methods, results, and conclusions	2
	**Introduction**	
3	**Problem formulation** - Description and significance of the problem/phenomenon studied; review of relevant theory and empirical work; problem statement	3
4	**Purpose or research question** - Purpose of the study and specific objectives or questions	3
	**Methods**	
5	**Qualitative approach and research paradigm** - Qualitative approach (e.g., ethnography, grounded theory, case study, phenomenology, narrative research) and guiding theory if appropriate; identifying the research paradigm (e.g., postpositivist, constructivist/ interpretivist) is also recommended; rationale**	4 - 5
6	**Researcher characteristics and reflexivity** - Researchers' characteristics that may influence the research, including personal attributes, qualifications/experience, relationship with participants, assumptions, and/or presuppositions; potential or actual interaction between researchers' characteristics and the research questions, approach, methods, results, and/or transferability	4
7	**Context** - Setting/site and salient contextual factors; rationale**	4
8	**Sampling strategy** - How and why research participants, documents, or events were selected; criteria for deciding when no further sampling was necessary (e.g., sampling saturation); rationale**	4 - 5
9	**Ethical issues pertaining to human subjects** - Documentation of approval by an appropriate ethics review board and participant consent, or explanation for lack thereof; other confidentiality and data security issues	11
10	**Data collection methods** - Types of data collected; details of data collection procedures including (as appropriate) start and stop dates of data collection and analysis, iterative process, triangulation of sources/methods, and modification of procedures in response to evolving study findings; rationale**	4 - 5
11	**Data collection instruments and technologies** - Description of instruments (e.g., interview guides, questionnaires) and devices (e.g., audio recorders) used for data collection; if/how the instrument(s) changed over the course of the study	4
12	**Units of study** - Number and relevant characteristics of participants, documents, or events included in the study; level of participation (could be reported in results)	Included in results
13	**Data processing** - Methods for processing data prior to and during analysis, including transcription, data entry, data management and security, verification of data integrity, data coding, and anonymization/de-identification of excerpts	4 - 5
14	**Data analysis** - Process by which inferences, themes, etc., were identified and developed, including the researchers involved in data analysis; usually references a specific paradigm or approach; rationale**	4 – 5

For peer review only

15	**Techniques to enhance trustworthiness** - Techniques to enhance trustworthiness and credibility of data analysis (e.g., member checking, audit trail, triangulation); rationale**	10
	**Results/findings**	
16	**Synthesis and interpretation** - Main findings (e.g., interpretations, inferences, and themes); might include development of a theory or model, or integration with prior research or theory	5 – 8
17	**Links to empirical data** - Evidence (e.g., quotes, field notes, text excerpts, photographs) to substantiate analytic findings	5 – 8
	**Discussion**	
18	**Integration with prior work, implications, transferability, and contribution(s) to the field -** Short summary of main findings; explanation of how findings and conclusions connect to, support, elaborate on, or challenge conclusions of earlier scholarship; discussion of scope of application/generalizability; identification of unique contribution(s) to scholarship in a discipline or field	8 – 10
19	**Limitations** - Trustworthiness and limitations of findings	10
	**Other**	
20	**Conflicts of interest** - Potential sources of influence or perceived influence on study conduct and conclusions; how these were managed	10
21	**Funding** - Sources of funding and other support; role of funders in data collection, interpretation, and reporting	11

*The authors created the SRQR by searching the literature to identify guidelines, reporting standards, and critical appraisal criteria for qualitative research; reviewing the reference lists of retrieved sources; and contacting experts to gain feedback. The SRQR aims to improve the transparency of all aspects of qualitative research by providing clear standards for reporting qualitative research.

**The rationale should briefly discuss the justification for choosing that theory, approach, method, or technique rather than other options available, the assumptions and limitations implicit in those choices, and how those choices influence study conclusions and transferability. As appropriate, the rationale for several items might be discussed together.

Citation:

O'Brien, Bridget C. PhD; Harris, Ilene B. PhD; Beckman, Thomas J. MD; Reed, Darcy A. MD, MPH; Cook, David A. MD, MHPE Standards for Reporting Qualitative Research, Academic Medicine: September 2014 - Volume 89 - Issue 9 - p 1245-1251 doi: 10.1097/ACM.0000000000000388.

For peer review only

BMJ Open

ATTITUDES AND ADHERENCE TO CHANGES IN NUTRITION AND PHYSICAL ACTIVITY FOLLOWING SURGERY FOR PROSTATE CANCER: A QUALITATIVE STUDY

Journal:	*BMJ Open*
Manuscript ID	bmjopen-2021-055566.R2
Article Type:	Original research
Date Submitted by the Author:	20-May-2022
Complete List of Authors:	Robles, Luke; University of Bristol, National Institute for Health Research Bristol Biomedical Research Centre, University Hospitals Bristol and Weston NHS Foundation Trust and University of Bristol<br>Shingler, Ellie; University of Bristol, National Institute for Health Research Bristol Biomedical Research Centre, University Hospitals Bristol and Weston NHS Foundation Trust and University of Bristol<br>McGeagh, Lucy ; Oxford Brookes University, Supportive Cancer Care Research Group, Faculty of Health and Life Sciences, Oxford Institute of Nursing, Midwifery and Allied Health Research; University of Bristol, National Institute for Health Research Bristol Biomedical Research Centre, University Hospitals Bristol and Weston NHS Foundation Trust and University of Bristol<br>Rowe, Edward; North Bristol NHS Trust, Bristol Urology Institute, Department of Urology<br>Koupparis, Anthony; North Bristol NHS Trust, Bristol Urology Institute, Department of Urology<br>Bahl, Amit; University Hospitals Bristol and Weston NHS Foundation Trust, Bristol Haematology and Oncology Centre<br>Shiridzinomwa, Constance; North Bristol NHS Trust, Clinical Research Centre<br>Persad, Raj; North Bristol NHS Trust, Bristol Urology Institute, Department of Urology<br>Martin, Richard; University of Bristol, Bristol Medical School, Population Health Sciences; University of Bristol, National Institute for Health Research Bristol Biomedical Research Centre, University Hospitals Bristol and Weston NHS Foundation Trust and University of Bristol<br>Lane, Athene; University of Bristol, Bristol Medical School, Population Health Sciences; University of Bristol, National Institute for Health Research Bristol Biomedical Research Centre, University Hospitals Bristol and Weston NHS Foundation Trust and University of Bristol
**Primary Subject Heading**:	Qualitative research
Secondary Subject Heading:	Urology
Keywords:	Prostate disease < UROLOGY, Nutritional support < ONCOLOGY, QUALITATIVE RESEARCH

SCHOLARONE™
Manuscripts

I, the Submitting Author has the right to grant and does grant on behalf of all authors of the Work (as defined in the below author licence), an exclusive licence and/or a non-exclusive licence for contributions from authors who are: i) UK Crown employees; ii) where BMJ has agreed a CC-BY licence shall apply, and/or iii) in accordance with the terms applicable for US Federal Government officers or employees acting as part of their official duties; on a worldwide, perpetual, irrevocable, royalty-free basis to BMJ Publishing Group Ltd ("BMJ") its licensees and where the relevant Journal is co-owned by BMJ to the co-owners of the Journal, to publish the Work in this journal and any other BMJ products and to exploit all rights, as set out in our licence.

The Submitting Author accepts and understands that any supply made under these terms is made by BMJ to the Submitting Author unless you are acting as an employee on behalf of your employer or a postgraduate student of an affiliated institution which is paying any applicable article publishing charge ("APC") for Open Access articles. Where the Submitting Author wishes to make the Work available on an Open Access basis (and intends to pay the relevant APC), the terms of reuse of such Open Access shall be governed by a Creative Commons licence – details of these licences and which Creative Commons licence will apply to this Work are set out in our licence referred to above.

Other than as permitted in any relevant BMJ Author's Self Archiving Policies, I confirm this Work has not been accepted for publication elsewhere, is not being considered for publication elsewhere and does not duplicate material already published. I confirm all authors consent to publication of this Work and authorise the granting of this licence.

For peer review only

ATTITUDES AND ADHERENCE TO CHANGES IN NUTRITION AND PHYSICAL ACTIVITY FOLLOWING SURGERY FOR PROSTATE CANCER: A QUALITATIVE STUDY

AUTHORS

Corresponding author
Professor J. Athene Lane
Canynge Hall, 39 Whatley Road, Bristol, BS8 2PS
Athene.Lane@bristol.ac.uk

To appear in the order shown below.
* Authors contributed equally.

Dr Luke A Robles
NIHR Bristol Biomedical Research Centre (Nutrition, Diet and Lifestyle theme), University Hospitals Bristol and Weston NHS Foundation Trust and University of Bristol, Bristol, UK

Mrs Ellie Shingler
NIHR Bristol Biomedical Research Centre (Nutrition, Diet and Lifestyle theme), University Hospitals Bristol and Weston NHS Foundation Trust and University of Bristol, Bristol, UK

Dr Lucy McGeagh
Supportive Cancer Care Research Group, Faculty of Health and Life Sciences, Oxford Institute of Nursing, Midwifery and Allied Health Research, Oxford Brookes University, Oxford, UK
NIHR Bristol Biomedical Research Centre (Nutrition, Diet and Lifestyle theme), University Hospitals Bristol and Weston NHS Foundation Trust and University of Bristol, Bristol, UK

Mr Edward Rowe
Bristol Urology Institute, Department of Urology, North Bristol NHS Trust, Bristol, UK

Mr Anthony Koupparis
Bristol Urology Institute, Department of Urology, North Bristol NHS Trust, Bristol, UK

Professor Amit Bahl
Bristol Haematology and Oncology Centre, University Hospitals Bristol and Weston NHS Foundation Trust, Bristol, UK

Mrs Constance Shiridzinomwa
Clinical Research Centre, North Bristol NHS Trust, Bristol, UK

Professor Raj Persad
Bristol Urology Institute, Department of Urology, North Bristol NHS Trust, Bristol, UK

Professor Richard M Martin*
Bristol Medical School, Population Health Sciences, University of Bristol, Bristol, UK
NIHR Bristol Biomedical Research Centre (Nutrition, Diet and Lifestyle theme), University Hospitals Bristol and Weston NHS Foundation Trust and University of Bristol, Bristol, UK

Professor J. Athene Lane*
Bristol Medical School, Population Health Sciences, University of Bristol, Bristol, UK
NIHR Bristol Biomedical Research Centre (Nutrition, Diet and Lifestyle theme), University Hospitals Bristol and Weston NHS Foundation Trust and University of Bristol, Bristol, UK

Word count: 4357

For peer review only

For peer review only - http://bmjopen.bmj.com/site/about/guidelines.xhtml

KEYWORDS

Prostate cancer, adherence, nutrition, physical activity

ABSTRACT

Objectives

Interventions designed to improve men's diet and physical activity (PA) have been recommended as methods of cancer prevention. However, little is known about specific factors that support men's adherence to these health behaviour changes, which could inform theory-led diet and PA interventions. We aimed to explore these factors in men following prostatectomy for prostate cancer (PCa).

Design, setting and participants

A qualitative study using semi-structured interviews with men, who made changes to their diet and/or PA as part of a factorial randomised controlled trial conducted at a single hospital in South West England. Participants were 17 men aged 66 years, diagnosed with localised PCa, and underwent prostatectomy. Interview transcripts underwent thematic analysis.

Results

Men were ambivalent about the relationship of nutrition and PA on PCa risk. They believed their diet and level of PA were reasonable before being randomised to their interventions. Men identified several barriers and facilitators to performing these new behaviours. Barriers included tolerance to dietary changes, PA limitations, and external obstacles. Facilitators included partner involvement in diet, habit formation, and brisk walking as an individual activity. Men discussed positive effects associated with brisk walking, such as feeling healthier, but not with nutrition interventions.

Conclusions

The facilitators to behaviour change suggest that adherence to trial interventions can be supported using well-established behaviour change models. Future studies may benefit from theory-based interventions to support adherence to diet and PA behaviour changes in men diagnosed with PCa.

ARTICLE SUMMARY

- This study provided a thematic analysis of men making diet and PA changes soon after prostatectomy, which included a negative case analysis to support the rigour of the study.
- The study included a small sample size of 17 men.
- Data analysis was limited by the lack of depth in men's responses which is likely due to interviews being part of the data collection for a feasibility randomised controlled trial.
- All men, except one, were white indicating that the study sample was not representative of the patient population.

BACKGROUND

Prostate cancer (PCa) is the most common form of cancer in men in the UK with over 48,000 new diagnoses every year [1]. Established risk factors are increasing age, ethnicity (black African or Caribbean), and a family history of PCa [2]. Modifiable factors, such as nutrition and physical activity (PA), have also been linked to PCa risk and progression [3, 4]. A higher intake of cruciferous vegetables (e.g., cabbage, cauliflower) is

For peer review only - http://bmjopen.bmj.com/site/about/guidelines.xhtml

associated with a reduction in PCa incidence and progression [5, 6]. Lycopene, a carotenoid found in many brightly coloured fruit and vegetables, has been linked with reduced risk of cancer progression post-diagnosis [7]. High intakes of dairy products is also associated with increased PCa risk [8]. With regard to PA, observational studies suggest that moderate to vigorous PA is associated with reduced risk of PCa-specific mortality and biochemical recurrence. More specifically, three hours of moderate to vigorous PA per week is associated with a 61% decrease in PCa mortality compared with less than one hour [9]. The increase of PA on lower risk of PCa-specific mortality and recurrence is supported by intervention studies [10]. In addition, PA has been shown to reduce adverse effects of treatment and improve quality of life, particular in men receiving androgen derivation therapy [11, 12].

The World Cancer Research Fund recommends making changes to nutrition and PA behaviours as methods of cancer prevention [13]. Such behaviour changes include maintaining a plant-based diet (PBD) (i.e. consuming more grains, beans [14], five fruit and vegetables a day [15], and to performing 30 minutes of moderate to vigorous PA a day and limiting sedentary behaviours [16], and the use of supplements, such as lycopene [17]. However, evidence has shown that most cancer survivors do not meet these recommendations. For example, Blanchard and colleagues [18] reported that, out of over 2000 PCa survivors, only 43% were meeting the recommendations for fruit and vegetable consumption and only 16% were meeting the recommendations for PA.

A systematic review [19] reported that nutrition interventions for cancer populations are rarely guided by behaviour theory. However, theory-based interventions were most effective at improving nutrition changes over a median follow-up of 12 months. There is limited evidence on psychological and behavioural factors that support adherence to nutrition interventions for men with PCa [20]. Furthermore, previous PA intervention studies with patients with chronic conditions, including cancer, have identified several factors that could support adherence to PA [21]. However, few of these studies have explored the psychological and behavioural factors which could align with exist models of behaviour change to enhance PA intervention in men undergoing prostatectomy. For example, a narrative review of behaviour change theories used in PA interventions in urological cancer survivors reported constructs of the Theory of Planned Behaviour (TPB) and the Trans-theoretical Model have been shown to increase men's motivation to be more physical active either during or following PCa treatment [22].

Our qualitative study aimed to identify factors associated with adherence to diet and PA interventions in men following prostatectomy for localised (organ-confined) PCa, which could inform such theory-led interventions in this patient population.

METHOD

This descriptive qualitative study was part of a factorial randomised controlled trial (RCT), Prostate cancer Evidence of Exercise and Nutrition Trial (PrEvENT) [23, 24], conducted at a single hospital in South West England. This trial assessed the feasibility and acceptability of nutritional and PA interventions for men after prostatectomy for localised PCa. Details of the trial can be found elsewhere (ISRCTN99048944) [23]. In brief, men were randomly allocated to nutritional and/or PA interventions (Table 1). This study was written in accordance with the Standards for Reporting Qualitative Research recommendations [25].

For peer review only - http://bmjopen.bmj.com/site/about/guidelines.xhtml

Table 1. Nutritional and physical activity interventions

Intervention	Allocation[†]	Description
Nutritional	Plant-based diet	• 5 fruit and vegetables per day<br>• Substitute dairy milk for non-dairy alternative (e.g., soya, almond or rice milk)
	Lycopene supplementation	• 10mg lycopene capsule taken once per day
	Control	• No changes to usual nutrition
Physical activity	Brisk walking	• 30 minutes brisk walking, 5 times per week
	Control	• No changes to usual daily physical

[†] Each participant was allocated to both a nutritional and physical activity intervention (factorial randomisation)

For peer review only

Participants

Seventeen men from the RCT, with an age range of 53 to 81 years (median = 66 years), were recruited into the qualitative element of the study having provided informed consent to be contacted regarding an interview. Purposive sampling was employed to ensure maximum variation across the intervention arms and to ensure that the sample consisted of various demographic characteristics such as age, employment status, and educational level [14]. Trial eligibility included men who were diagnosed with localised PCa, undergoing prostatectomy with no restrictions to performing the interventions. Twenty-five men were approached for interview in person during their 6 months research clinic visit appointment of the feasibility study. Six men were unable to attend due to external and personal circumstances (i.e., did not have the time during the clinic appointment (n=3), interviewer not available (n=2), and participant unwell (n=1)) and two men declined giving no reason. Seventeen men agreed and were interviewed. All men interviewed, except one man who was Caribbean, were reported as White British or White Other. Most men were retired (n=12) and married (n=13). Over half of the men were educated to secondary school level (n=9) (Table 2).

Table 2. Participant characteristics and intervention allocation

		n = 17	
		**n or median**	**% (range)**
Age (years)		66	(53-81)
Ethnicity	White British/White other	16	94
	Caribbean	1	6
Marital status	Married	13	76
	Not married	4	24
Education level	Secondary school (e.g., O-levels, GCSE)	9	53
	University	7	41
	Further education (e.g., A levels, HND)	1	6
Occupation status	Retired	12	71
	Employed	5	29
Intervention arm	Lycopene and brisk walking	4	23
	Lycopene and physical activity control	3	18
	Plant-based diet and brisk walking	3	18
	Plant-based diet and physical activity control	3	18
	Brisk walking and nutritional control	3	18
	Control	1	6

For peer review only - http://bmjopen.bmj.com/site/about/guidelines.xhtml

Data collection

Men took part in semi-structured interviews between April 2015 and May 2016 after completing their final 6-month follow-up. Interviews were conducted in-person within a private research clinic room (n=12). For those who were unable to attend in person, a telephone interview was arranged (n=5). Interviews were conducted by three authors (ES, n=9; LM, n=7; LR, n=1), whose backgrounds include Public Health (ES) and Health Psychology (LM, LR), and lasted between 19 to 84 minutes. All three authors were involved in the data collection process of PrEvENT, although had very minimal contact with participants. Interviews followed a pre-defined interview topic guide (Supplementary material 1), in which questions focused on participants' experiences of performing the interventions from a trial perspective. However, participant responses often included topics associated with long-term adherence to behaviour changes. One man in the control group was included in the sampling. He received no intervention aside from standard publicly available nutrition and PA information, if requested. Data from responses about his diet and PA before participation in the trial was only used for analysis.

Data analysis

Interviews were audio recorded and transcribed for analysis. The transcripts were checked against the audio recordings for accuracy. Data were analysed using inductive thematic analysis with the aid of NVivo 10 software [26]. This method of analysis was chosen with the aim of understanding participant experiences of making behaviour changes beyond those related to study processes of the RCT (e.g., feasibility outcomes). There were also no preconceptions about what themes would be identified from the data. Data analysis involved reading through the transcripts to increase familiarity with the data. They were, then, coded for items of data relating to the research question. The coding process was performed by one researcher (ES) and checked for consistency by a second researcher (LR). These codes were collated to form themes, which were reviewed and refined until a coherent narrative of the men's experiences was produced. Themes were reviewed and discussed regularly by both researchers to ensure they accurately represented the original data. A constant comparative approach was used to look at differences between sample characteristics, such as age, employment status, and intervention arm. Negative case analysis (i.e., identifying contradictory data) were used to broaden or confirm the interpretation of the themes and were resolved through discussion between the researchers and revisiting the transcripts. Data analysis was conducted throughout the data collection process to allow for initial themes to be explored in subsequent interviews. This also allowed researchers to decide when data saturation (i.e., no new themes or additional information emerged from the interviews) was reached.

Patient and Public Involvement (PPI)

A PCa PPI group were involved in the concept stages of PrEvENT and reviewed trial documentation, including the interview participant information sheet, consent form, and topic guide.

RESULTS

The analysis yielded five overarching themes: (1) causal beliefs about PCa; (2) perceptions of a healthy diet and PA before diagnosis; (3) barriers to adherence; (4) facilitators of adherence; and (5) perceived benefits of behaviour change. The thematic map is shown in Figure 1.

Causal beliefs about PCa

Men perceived that cancer was caused by external factors such as ageing, genetics, environment agents (i.e., radiation from nuclear sites). When asked about the impact of diet and PA with cancer, several men

For peer review only - http://bmjopen.bmj.com/site/about/guidelines.xhtml

believed there was little or no association. Men obtained information about PCa from media sources that were, at times, found to be conflicting.

"…I've looked at these things [causes of cancer] to some extent and I must admit that the evidence for diet-cancer links, to my view, has been weak." P6, PBD and brisk walking

"Well you read it in the paper and sometimes you think there might be [a link with cancer]." P8, lycopene and brisk walking

In contrast, a small number of men reported that they believed that healthy eating and regular exercise was associated with their cancer and this was one reason for maintaining a healthy diet and being physically active.

Interviewer: "…before you took part in the trial, had you ever thought about the links between your lifestyle, what you ate and how much activity you did and cancer?"

Participant: "Well I was concerned that it might be related so I have always tried to eat the right things and do exercise and walking so I just carried on as before. I didn't do any extra walking but I do try and walk at least two miles a day." P13, PBD

Perceptions of a healthy diet and PA before diagnosis

Men across all the intervention arms believed that they maintained a healthy diet before being diagnosed with PCa. However, the evidence for this notion was mixed. Some men described being able to effectively maintain a healthy diet.

"I really, sort of, eat a fairly Mediterranean diet. I use olive oil instead of butter, for example. If I have a sandwich or something, I put olive oil on. We cook all our own vegetables. I used to have an allotment, which I had to give up because of my leg, because of my knee." P5, lycopene and brisk walking

While other men described making extensive changes on starting the PBD within the trial.

"…I found that I was really, sort of, [toning] myself up almost on fruit and veg. I think it said you had to eat 5 more portions of fruit and veg a day than normal, so I was getting up to, at some stages, about 20, I think, a day." P17, PBD and brisk walking

Men generally described themselves as participating in daily PA, such as going out for regular walks, before being diagnosed. Some men also belonged to a gym.

"I tend to do stretching exercises every day and I do a lot of gardening as well. I love gardening and I do walk. As I say, I've got two little terriers." P15, lycopene

"I'm quite active anyway. Even beforehand I'd get a bike ride, a good two hour or so bike ride once a week and a gym session and once or twice round that walk anyway or sometimes longer. It wasn't a complete change of lifestyle for me." P14, lycopene and brisk walking

For peer review only - http://bmjopen.bmj.com/site/about/guidelines.xhtml

Barriers to adherence

Tolerance of the interventions

Few obstacles were identified by men regarding their ability to adherence to the interventions. Men with co-morbidities, including knee pain, were restricted from walking 'briskly' as this was found to aggravate their physical conditions. Some men struggled to adhere to soya milk, mostly due to its taste in coffee or tea. One man believed lycopene caused him some constipation and, therefore, he preferred not to consume his supplements in the long-term.

Interviewer: "If we said, "Can you do this for 12 months?" Could you have carried on?"

Participant: "I would have done yes, but as choice I would say no. I do believe that it causes me slight constipation so I would rather not." P10, lycopene

External obstacles

Most men relied on good weather. There was also little motivation to walk elsewhere when the weather was bad.

"…there were some day where it was a total wash-out, and you think, "Well, there's no point in even trying," you know. "I'll make this my quiet day"…" P3, brisk walking and lycopene

There were also clear differences in men's perceived ability to adhere to brisk walking between those employed and those retired. Work was described as affecting men's success at maintaining their brisk walking.

"Anyway, before I went back to work, it was easy to discipline myself to say, "Right, I'm going to go walking in the morning and in the afternoon, twice a day," but when I went back to work, that wasn't so easy." P6, PBD and brisk walking

"I did think if I'd been working, especially over the winter, it would have been quite difficult to do because you get up and go into work in the dark and come home in the dark." P14, lycopene and brisk walking

Activities that intervened with men's usual routine, such as going on holiday, eating out, and staying with friends were reported to affect some of the men's adherence to both the PBD and brisk walking interventions.

"… I went to my son's [place] and they don't eat a lot of fruit and vegetables there right now, so perhaps for a couple of days then, it was a low count." P2, PBD

"… we were travelling, visiting friends and doing things, so there were some days there when I just couldn't do any walking." P1, brisk walking

Facilitators of adherence

Partner involvement

Men often suggested that their wives or partners would frequently prepare their meals and this would help them with their adherence to the PBD, especially if they also consumed a diet high in fruit and vegetables.

For peer review only - http://bmjopen.bmj.com/site/about/guidelines.xhtml

For peer review only

"She is wonderful and she looks after me absolutely, 100%, our food is ready by six…My wife is a three veg, four veg, five veg and, she is greens, she thinks they are wonderful." P2, PBD

Habit formation

Five out of the six men in the lycopene arm were on prescribed medication for other health conditions. They suggested that the routine of self-medicating meant that they found it easy to adhere to taking the supplements.

"It becomes very easy, because the Lycopene, I took every morning with my hypertension medication and it just became part of the breakfast …" P3, lycopene and brisk walking

A couple of brisk walking men, who were physically active and belonged to a gym prior to initiating behaviour changes for the purpose of the trial mentioned that they would overcome barriers, such as bad weather, by incorporating it as part of their usual indoor exercise routine.

"…I built my walk into the gym routine. I did 30 minutes on a treadmill sitting at about 6 kph or something like that with grading…" P14, lycopene and brisk walking

Brisk walking as individual activity

Although attempts were made by men to carry out their brisk walk with others, most men claimed that they were happy to walk by themselves and were not dependent on others to help motivate them. Men discussed that one of the reasons why they brisk walked by themselves was due to its intensity (i.e., walking at a pace where they could talk but not sing) as they felt others were not able to walk at the same pace.

"My wife has joined a walking group, but they don't go fast enough. It was too much of an amble. She doesn't walk very fast, by comparison. If ever we're going anywhere, I have to modify my pace to suit her. It was better to do it on my own." P1, brisk walking

Perceived benefits of behaviour change

Most men reported there were many benefits to being more physically active. Several men discussed that going out for a walk provided them with a structured way of performing a reasonable level of PA, which they would not normally do.

"I think, I mean, although I've painted a picture of being quite active, then you know, I mean, it wasn't very organised, you know what I mean? What this did was to impose a routine on me, which I was quite happy with. And it's like setting a goal, isn't it?" P3, lycopene and brisk walking

It also gave them a sense of feeling healthier. One man spoke about how walking to work enabled him to 'clear his head' before starting work. Another man even associated his brisk walking to success of subsequent radiotherapy.

"The walking because it kept me a bit healthier and fitter I think I did better on the radiotherapy." P8, lycopene and brisk walking

Men did not comment on the physical or psychological outcomes they experienced from consuming more fruit and vegetables or lycopene despite knowing the potential health benefits.

For peer review only - http://bmjopen.bmj.com/site/about/guidelines.xhtml

For peer review only

DISCUSSION

Summary of findings

We aimed to explore factors influencing adherence to nutrition and PA interventions in men, who had prostatectomy following a diagnosis of localised PCa. Our findings showed that men believed their cancer was caused by external factors, such as age and genetics. They discussed eating healthily and regularly exercising before their diagnosis and barriers and facilitators to their behaviour changes. Overall, men found the PA intervention beneficial to their health and wellbeing.

Support with other studies

Men were not fully convinced that cancer was caused or related to their nutrition or PA. They attributed the cause of their cancer to external factors including age and genetic factors. These findings are supported by another qualitative study [27], which shown that PCa survivors can overestimate the significance of environmental factors, such as pollution and stress, and underestimate behaviour factors associated with increased cancer risk, such as obesity and inactivity. In contrast, observational evidence showed that a high proportion of women attributed diet (68%) to their breast cancer diagnosis in addition to external factors (i.e. hormones) [28]. Furthermore, in a sample of 40 men interviewed about their lifestyle behaviours following their PCa diagnosis, 60% were obese and 88% were not motivated to change their smoking, alcohol and/or their eating behaviour [29]. These findings could be indicative of men's preference to believe in causal factors that are outside their control, and reinforce the importance of lifestyle interventions at the time of diagnosis.

Men from all the intervention groups believed that they adhered well to their nutrition intervention. While some men followed the intervention guidelines, others made quite extreme changes to their diet, such as eating well-over the recommended daily intake of fruit and vegetables. This suggests that men may benefit from more education on eating practices, including more detail on portion sizes.

Men's tolerance to changes in their diet impacted on their adherence to their nutrition interventions. Some men did not like the taste of soya milk and reverted to dairy milk or alternatives forms of diary free milk. This somewhat contradicts findings from previous trials that have shown men to adhere well to a daily consumption of soya products over significant follow-ups. However, these trials incorporated soya products in the form of drink supplements [30, 31] and soya-bread [32]. Thus, the way in which soya products are consumed could influence how men adhere to these products in the long-term. With regard to lycopene, its side-effects are not well-known although other PCa trials have reported diarrhoea and flatulence as plausible side-effects of the supplement in few cases [33]. One man did believe that the constipation he experienced during the trial was due to taking lycopene. Therefore, constipation could potentially be a side-effect and men would need to be aware of these effects and advised how to manage them in future trials.

Barriers to brisk walking included weather conditions and a lack of time. These barriers to regular walking have been cited by prostate and other patient populations [29] [34] [35]. Men were assessed for co-morbidities prohibiting them from performing brisk walking before entering PrEvENT. Therefore, it could be speculated that the physical restrictions to brisk walking reported by men are indicative of their motivation to brisk walk when obstacles arise.

Partners were found to be significantly involved in choosing and preparing meals for men. Partners are often involved at each stage of men's treatment pathway, including helping them comply with pre-prostatectomy preparation, such as improving fitness and losing weight [36]. Thus, this finding suggests that men would adhere better to PBD interventions with partner involvement. In contrast, men discussed the PA intervention as one which they preferred to do by themselves. This finding somewhat contradicts evidence

For peer review only - http://bmjopen.bmj.com/site/about/guidelines.xhtml

which has shown men to report physical, mental and relationship benefits from PA interventions involving their partners [37]. However, the prescribed nature of couple-based interventions is likely to attribute to its efficacy.

A facilitator to the lycopene intervention was men consuming the supplement along with their existing medication regime. In a similar vein, men who were exercising regularly, before being enrolled in the RCT, were able to include brisk walking into their exercise schedule. Evidence from a previous RCT suggested that men's exercise adherence was more difficult for those who had not considered exercising before entering the trial [38]. These facilitators for lycopene and brisk walking adherence suggest adherence is linked to habitual behaviours (i.e., actions to contextual cues). Habitual formation behaviours have been shown to increase adherence to both nutrition and PA interventions. The current findings suggest incorporating new health behaviours with existing healthy habits could strength adherence [39].

Physical and psychological benefits were reported by those men who brisk walked. These beneficial effects have been reported in a prospective study measuring PA in men with PCa [40]. Men who adhered to 150 minutes of moderate PA post-diagnosis had significantly better physical ($\beta = 6.01$, 95% confidence interval (CI): 4.15 to 7.86) and mental ($\beta = 2.32$; 95% CI: 0.29-4.34) quality of life (i.e., physical functioning and better mood states) compared to men who were non-adherent. Such physical and psychological outcomes have been reported as facilitators to exercise [29] and have the potential to help men adhere well to their brisk walking.

Strengths and limitations

The strength of this study is that it has provided a thematic analysis of men making diet and PA changes soon after prostatectomy, which included a negative case analysis to support the rigour of the study. However, this study has several limitations. Data analysis was limited by the lack of depth in responses from the interviews. This is likely due to the interviews being part of data collection for a feasibility RCT, which assessed trial processes as well as intervention adherence. Therefore, further assessments of rigour would not have benefitted the data analysis. The sample size was small (n=17) and all men, except one, were white and the majority were married. It is unlikely that the data fully represents the experiences of men from other ethnic groups or single men without support from partners with their intervention. In addition, men in all the intervention arms discussed that they were already maintaining a healthy diet and engaging in regular physical activities before their diagnosis. This could suggest that the current findings are limited to men more willing and able to perform these health behaviours. As this is a qualitative study, findings are based on subjective accounts of behaviour change and there is the chance of men over reporting areas of their behaviour change due to recall bias and men wanted to please the researchers [41].

Main implications and future research

Intervention studies should embrace the use of social support to reinforce adherence to dietary changes, especially with PBD interventions where partners are involved with meals prepared at home. Behavioural interventions that can be performed with existing behaviours (e.g., medication regime) are likely to increase participants' confidence and adherence. Further work may want to tailor interventions that consider contextual cues and one's belief in the ability to perform the desired behaviour, as well as behavioural strategies that support adherence. A theory-led behavioural model can both guide and assist with evaluating interventions [42]. Our study findings indicate that men are motivated to make changes to their diet and level of PA following prostatectomy. However, men's motivation was not related to beliefs that diet and PA was associated with their PCa. Other psychological factors could explain men's motivation to adherence to these behaviour changes, such as symptom control, which could be explored using qualitative studies. Barriers to adhering to their behaviour changes related to physical (i.e., weather, time) and social opportunities (e.g., going on holiday). These findings suggest that future nutrition and PA interventions

For peer review only - http://bmjopen.bmj.com/site/about/guidelines.xhtml

guided by a behavioural model, which help identify these barriers and incorporate techniques such as problem-solving, will improve adherence [43, 44]. The COM-B model [45] could be one that is suitable for this patient population. This model proposes that a person's motivation to perform and maintain a behaviour is supported by their capability (i.e., psychologically and physically) and opportunity (i.e., social and physical) to perform the behaviour. Future studies may consider exploring the use of this model in nutrition and PA intervention studies with PCa populations.

CONCLUSION

The findings from this study may be helpful in developing and implementing future nutrition and PA interventions in men after receiving prostatectomy for PCa. This qualitative study suggests that behaviour change models could support adherence to nutritional and PA behaviours.

ACKNOWLEDGEMENTS

The authors would like to thank all participants for taking part in the qualitative interviews. The authors would also like to thank Dr Aidan Searle for his advice on the qualitative analysis of this study.

COMPETING INTERESTS

None declared.

FUNDING

This study was funded by the NIHR Biomedical Research Centre at University Hospitals Bristol and Weston NHS Foundation Trust and the University of Bristol. The views expressed are those of the author(s) and not necessarily those of the NIHR or the Department of Health and Social Care. The funder was not involved in the design of the study and collection, analysis, and interpretation of data and in writing the manuscript.

ETHICS APPROVAL AND CONSENT TO PARTICIPATE

PrEvENT received Research Ethics Committee approval from the National Research Ethics Service Committee South West – Cornwall & Plymouth on 8 April 2014 (REC ref 14/SW/0056). All participants provided written informed consent on enrolment into the trial. Additional oral consent was obtained from participants who took part in telephone interviews. A participant information sheet and consent form were sent in advance of the telephone interviews.

CONSENT FOR PUBLICATION

Not applicable.

DATA AVAILABILITY STATEMENT

The datasets used and/or analysed during the current study is available from the corresponding author on reasonable request.

AUTHOR CONTRIBUTIONS

ES and LHM collected the data. LR, ES, and LHM analysed and interpreted the data and were major contributors to the manuscript. LHM, AB, RP, JAL, and RMM conceived the study, reviewed the article, and

For peer review only - http://bmjopen.bmj.com/site/about/guidelines.xhtml

critically revised the manuscript. ER, AK, AB, CS, and RP were involved in the study conduct and critically revised the manuscript. All authors read and approved the final manuscript.

REFERENCES

1. Cancer Research UK. Prostate cancer incidence statistics: Cancer Research UK; 2017 [Available from: https://www.cancerresearchuk.org/health-professional/cancer-statistics/statistics-by-cancer-type/prostate-cancer/incidence#heading-Zero.
2. Prostate Cancer UK. Who is at risk? : Prostate Cancer UK; [Available from: https://prostatecanceruk.org/prostate-information/are-you-at-risk#black-men.
3. Stein CJ, Colditz GA. Modifiable risk factors for cancer. British journal of cancer. 2004;90(2):299-303.
4. Peisch SF, Van Blarigan EL, Chan JM, Stampfer MJ, Kenfield SA. Prostate cancer progression and mortality: a review of diet and lifestyle factors. World journal of urology. 2016.
5. Richman EL, Carroll PR, Chan JM. Vegetable and fruit intake after diagnosis and risk of prostate cancer progression. International journal of cancer. 2012;131(1):201-10.
6. Liu B, Mao Q, Cao M, Xie L. Cruciferous vegetables intake and risk of prostate cancer: a meta-analysis. International journal of urology : official journal of the Japanese Urological Association. 2012;19(2):134-41.
7. Chan JM, Holick CN, Leitzmann MF, Rimm EB, Willett WC, Stampfer MJ, et al. Diet after diagnosis and the risk of prostate cancer progression, recurrence, and death (United States). Cancer causes & control : CCC. 2006;17(2):199-208.
8. López-Plaza B, Bermejo LM, Santurino C, Cavero-Redondo I, Álvarez-Bueno C, Gómez-Candela C. Milk and Dairy Product Consumption and Prostate Cancer Risk and Mortality: An Overview of Systematic Reviews and Meta-analyses. Advances in Nutrition. 2019;10(suppl_2):S212-S23.
9. Kenfield SA, Stampfer MJ, Giovannucci E, Chan JM. Physical activity and survival after prostate cancer diagnosis in the health professionals follow-up study. Journal of clinical oncology : official journal of the American Society of Clinical Oncology. 2011;29(6):726-32.
10. Cormie P, Zopf EM, Zhang X, Schmitz KH. The Impact of Exercise on Cancer Mortality, Recurrence, and Treatment-Related Adverse Effects. Epidemiologic Reviews. 2017;39(1):71-92.
11. Bressi B, Cagliari M, Contesini M, Mazzini E, Bergamaschi FAM, Moscato A, et al. Physical exercise for bone health in men with prostate cancer receiving androgen deprivation therapy: a systematic review. Support Care Cancer. 2021;29(4):1811-24.
12. Cagliari M, Bressi B, Bassi MC, Fugazzaro S, Prati G, Iotti C, et al. Feasibility and Safety of Physical Exercise to Preserve Bone Health in Men with Prostate Cancer Receiving Androgen Deprivation Therapy: A Systematic Review. Phys Ther. 2021.
13. WCRF/AIRC. Food, nutrition, physical activity and the prevention of cancer: a global perspective Washington, DC: AICR; 2007 [Available from: https://www.wcrf.org/diet-and-cancer/.
14. Parsons JK, Newman VA, Mohler JL, Pierce JP, Flatt S, Marshall J. Dietary modification in patients with prostate cancer on active surveillance: a randomized, multicentre feasibility study. BJU international. 2008;101(10):1227-31.
15. Demark-Wahnefried W, Peterson B, McBride C, Lipkus I, Clipp E. Current health behaviors and readiness to pursue life-style changes among men and women diagnosed with early stage prostate and breast carcinomas. Cancer. 2000;88(3):674-84.
16. Hackshaw-McGeagh LE, Perry RE, Leach VA, Qandil S, Jeffreys M, Martin RM, et al. A systematic review of dietary, nutritional, and physical activity interventions for the prevention of prostate cancer progression and mortality. Cancer causes & control : CCC. 2015;26(11):1521-50.
17. Bunker CH, McDonald AC, Evans RW, de la Rosa N, Boumosleh JM, Patrick AL. A randomized trial of lycopene supplementation in Tobago men with high prostate cancer risk. Nutrition and cancer. 2007;57(2):130-7.

For peer review only - http://bmjopen.bmj.com/site/about/guidelines.xhtml

18. Blanchard CM, Courneya KS, Stein K. Cancer survivors' adherence to lifestyle behavior recommendations and associations with health-related quality of life: results from the American Cancer Society's SCS-II. Journal of clinical oncology : official journal of the American Society of Clinical Oncology. 2008;26(13):2198-204.
19. Avery KN, Donovan JL, Horwood J, Lane JA. Behavior theory for dietary interventions for cancer prevention: a systematic review of utilization and effectiveness in creating behavior change. Cancer causes & control : CCC. 2013;24(3):409-20.
20. Stacey FG, James EL, Chapman K, Courneya KS, Lubans DR. A systematic review and meta-analysis of social cognitive theory-based physical activity and/or nutrition behavior change interventions for cancer survivors. Journal of Cancer Survivorship. 2015;9(2):305-38.
21. Collado-Mateo D, Lavín-Pérez AM, Peñacoba C, Del Coso J, Leyton-Román M, Luque-Casado A, et al. Key Factors Associated with Adherence to Physical Exercise in Patients with Chronic Diseases and Older Adults: An Umbrella Review. International Journal of Environmental Research and Public Health. 2021;18(4):2023.
22. Rammant E, Bultijnck R, Caperchione CM, Trinh L. The Use of Theory to Develop Physical Activity Interventions in Urological Cancer Survivors: A Narrative Review. Seminars in Oncology Nursing. 2021;37(1):151109.
23. Hackshaw-McGeagh L, Lane JA, Persad R, Gillatt D, Holly JM, Koupparis A, et al. Prostate cancer - evidence of exercise and nutrition trial (PrEvENT): study protocol for a randomised controlled feasibility trial. Trials. 2016;17(1):123.
24. Shingler E, Hackshaw-McGeagh L, Robles L, Persad R, Koupparis A, Rowe E, et al. The feasibility of the Prostate cancer: Evidence of Exercise and Nutrition Trial (PrEvENT) dietary and physical activity modifications: a qualitative study. Trials. 2017;18(1):1-10.
25. O'Brien BC, Harris IB, Beckman TJ, Reed DA, Cook DA. Standards for reporting qualitative research: a synthesis of recommendations. Acad Med. 2014;89(9):1245-51.
26. Ritchie J, Spencer L. Qualitative data analysis for applied policy research. The qualitative researcher's companion. 2002;573:305-29.
27. Wold KS, Byers T, Crane LA, Ahnen D. What do cancer survivors believe causes cancer? (United States). Cancer Causes & Control. 2005;16(2):115-23.
28. Costanzo ES, Lutgendorf SK, Roeder SL. Common-sense beliefs about cancer and health practices among women completing treatment for breast cancer. Psychooncology. 2011;20(1):53-61.
29. Bressi B, Iotti C, Cagliari M, Fugazzaro S, Cavuto S, Bergamaschi FAM, et al. Physical exercise habits, lifestyle behaviors, and motivation to change among men with prostate cancer: a cross-sectional study. Supportive Care in Cancer. 2022;30(6):5017-26.
30. Hamilton-Reeves JM, Rebello SA, Thomas W, Kurzer MS, Slaton JW. Effects of soy protein isolate consumption on prostate cancer biomarkers in men with HGPIN, ASAP, and low-grade prostate cancer. Nutrition and cancer. 2008;60(1):7-13.
31. Bosland MC, Kato I, Zeleniuch-Jacquotte A, Schmoll J, Enk Rueter E, Melamed J, et al. Effect of soy protein isolate supplementation on biochemical recurrence of prostate cancer after radical prostatectomy: a randomized trial. Jama. 2013;310(2):170-8.
32. Ahn-Jarvis JH, Clinton SK, Grainger EM, Riedl KM, Schwartz SJ, Lee ML, et al. Isoflavone pharmacokinetics and metabolism after consumption of a standardized soy and soy-almond bread in men with asymptomatic prostate cancer. Cancer prevention research (Philadelphia, Pa). 2015;8(11):1045-54.
33. Haseen F, Cantwell MM, O'Sullivan JM, Murray LJ. Is there a benefit from lycopene supplementation in men with prostate cancer? A systematic review. Prostate cancer and prostatic diseases. 2009;12(4):325-32.
34. Hackshaw-McGeagh LE, Sutton E, Persad R, Aning J, Bahl A, Koupparis A, et al. Acceptability of dietary and physical activity lifestyle modification for men following radiotherapy or radical prostatectomy for localised prostate cancer: a qualitative investigation. BMC Urology. 2017;17(1):94.

For peer review only

35. Rogerson MC, Murphy BM, Bird S, Morris T. "I don't have the heart": a qualitative study of barriers to and facilitators of physical activity for people with coronary heart disease and depressive symptoms. The international journal of behavioral nutrition and physical activity. 2012;9:140.
36. Kirby R, Holmes K, Amoroso P. SUPPORTING THE SUPPORTER: HELPING THE PARTNER OF PATIENTS NEWLY DIAGNOSED WITH PROSTATE CANCER. BJU international. 2010;105(11):1489-90.
37. Winters-Stone KM, Lyons KS, Beer TM, Skiba MB, Hung A. A pilot feasibility study of Exercising Together© during radiation therapy for prostate cancer: a dyadic approach for patients and spouses. Pilot and Feasibility Studies. 2021;7(1):216.
38. Courneya KS, Segal RJ, Reid RD, Jones LW, Malone SC, Venner PM, et al. Three independent factors predicted adherence in a randomized controlled trial of resistance exercise training among prostate cancer survivors. Journal of Clinical Epidemiology. 2004;57(6):571-9.
39. Gardner B, Lally P, Wardle J. Making health habitual: the psychology of 'habit-formation'and general practice. Br J Gen Pract. 2012;62(605):664-6.
40. Farris MS, Kopciuk KA, Courneya KS, McGregor SE, Wang Q, Friedenreich CM. Associations of Postdiagnosis Physical Activity and Change from Prediagnosis Physical Activity with Quality of Life in Prostate Cancer Survivors. Cancer epidemiology, biomarkers & prevention : a publication of the American Association for Cancer Research, cosponsored by the American Society of Preventive Oncology. 2017;26(2):179-87.
41. Breakwell GM, Hammond S, Fife-Schaw C. Research methods in psychology. Journal of Environmental Psychology. 1994;14(4):335.
42. Abraham C, Michie S. A taxonomy of behavior change techniques used in interventions. Health psychology : official journal of the Division of Health Psychology, American Psychological Association. 2008;27(3):379-87.
43. Bourke L, Doll H, Crank H, Daley A, Rosario D, Saxton JM. Lifestyle intervention in men with advanced prostate cancer receiving androgen suppression therapy: a feasibility study. Cancer epidemiology, biomarkers & prevention : a publication of the American Association for Cancer Research, cosponsored by the American Society of Preventive Oncology. 2011;20(4):647-57.
44. Craike MJ, Livingston PM, Botti M. An exploratory study of the factors that influence physical activity for prostate cancer survivors. Supportive Care in Cancer. 2011;19(7):1019-28.
45. Michie S, van Stralen MM, West R. The behaviour change wheel: a new method for characterising and designing behaviour change interventions. Implement Sci. 2011;6:42.

LEGEND

Figure 1: Thematic map of qualitative analysis (PA, physical activity)

For peer review only

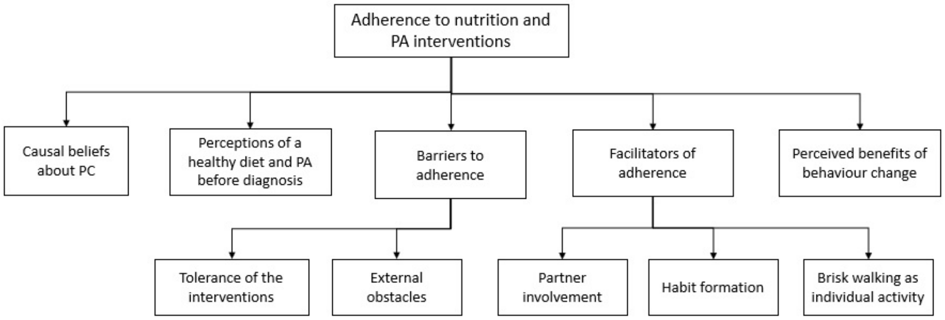

Figure 1: Thematic map of qualitative analysis (PA, physical activity)

247x104mm (96 x 96 DPI)

Patient Interview Topic Guide

PrEvENT: End of trial evaluation interview

Introduction

1. Can you tell me a little about your involvement in the trial?
   a) What arm were you allocated to?
   b) What exactly were you asked to do?

Intervention specifics

2. How would you describe your overall experience of taking part in the intervention?

3. What were the positives of…(intervention arm)

4. Were there any negative elements of…(intervention arm)

5. Would you change any elements of your intervention arm?
   a. Brisk walking for longer / shorter
   b. Add other supplements / take supplements less often
   c. Add more or less changes to diet
      i. If so, which / how?

6. How did you find the instructions you were given about your intervention arm and daily monitoring instructions?
   a. Could you suggest improvements to the instructions?

7. We asked you to make changes to your behaviour approximately 6 weeks after surgery; do you think this was too soon / not soon enough?
   a. What do you think would be the ideal time to make changes to behaviour?

8. How did you find wearing the PA monitoring tool?
   a. Were you able to wear it all of the time? If not, why not?
   b. When were you not able to wear the PA monitoring tool? Why not?
   c. What were barriers to wearing it?
   d. What were benefits to wearing it?
   e. What made it easier?
   f. How did you find the instructions that you were provided with for the monitor?
   g. Could you suggest improvements to the instructions?

9. If we had asked you to stay in the study for longer eg. carrying out the intervention, or completing questionnaires every 6 months, how would you feel about that?
   a. What would make that easier?

For peer review only - http://bmjopen.bmj.com/site/about/guidelines.xhtml

Behaviour change

10. How easy or difficult did you find making changes to your behaviour?
    a. Do you think this would have been different in a different arm? How?

11. Do you think you would be able to continue with these changes for a longer duration of time?

12. How easy or difficult did you find it to remember to carry out the intervention changes?
    a. Why was this?
    b. How did you remember / what advice could you give others to remember?

13. Did you talk to friends / family about the trial and the changes we asked you to make?
    a. How did they respond?
    b. Were they supportive? Critical? Sceptical?
    c. How did that affect you and your behaviours?

14. Have you seen or felt any benefits / negative effects from the changes we asked you to make?

15. Did you see any weight differences due to the change in diet or physical activity?
    a. Would that have made a difference to you?

16. Would you say you have made any lasting changes to your behaviour as a result of the research?
    a. What behavioural changes have you made?
    b. Why did you make these changes?
    c. Why do you plan to continue with these changes?

17. Has participating in the research made you think about your behaviours differently at all?

18. Do you plan to continue with the changes you made?
    a. If so, which? Why? Why not?

19. What are your opinions about the associations between diet, physical activity and cancer?
    a. What about the link with obesity?
    b. Would that make you reconsider your behaviours? What would make you reconsider them?

Trial logistics / contact / nurse appointments

20. How would you describe your experience of the trial form a logistic perspective?
    a. How the appointments were made?
    b. Ease of attending appointments?
    c. Length of appointments?

For peer review only

For peer review only - http://bmjopen.bmj.com/site/about/guidelines.xhtml

21. How did you find your research clinic appointments with the research nurse?
    a. Were these enjoyable?
    b. Did these affect your motivation to continue with the research?
    c. Were you provided with all of the information that you required?

22. How did you find the regular contact / reminders by the research nurse and research team?
    a. Which method did you find the most useful? Why?
    b. Did they affect your motivation?
    c. Do you think they helped you to remember / continue with the trial?

Overall experience

23. How would you sum up your overall experience of taking part in the trial?

24. How would you improve or change the trial to make it better for future participants?

25. Was participating different to what you had expected?
    a. In a positive / negative way?
    b. What had you expected?
    c. Had you participated in research prior to this?

26. Which elements of the trial did you enjoy the most?

27. Which elements of the trial did you not enjoy? Or enjoy the least?

28. Based on your experience in this trial, would you participate in research again?
    a. If so why/if not why not?

29. Final question, what made you agree to take part in the first place?

30. Is there anything further you wish to add?

For peer review only

For peer review only - http://bmjopen.bmj.com/site/about/guidelines.xhtml

Standards for Reporting Qualitative Research (SRQR)

Item no.	Topic/item	Page no.
	**Title and abstract**	
1	**Title** - Concise description of the nature and topic of the study Identifying the study as qualitative or indicating the approach (e.g., ethnography, grounded theory) or data collection methods (e.g., interview, focus group) is recommended	1
2	**Abstract** - Summary of key elements of the study using the abstract format of the intended publication; typically includes background, purpose, methods, results, and conclusions	2
	**Introduction**	
3	**Problem formulation** - Description and significance of the problem/phenomenon studied; review of relevant theory and empirical work; problem statement	3
4	**Purpose or research question** - Purpose of the study and specific objectives or questions	3
	**Methods**	
5	**Qualitative approach and research paradigm** - Qualitative approach (e.g., ethnography, grounded theory, case study, phenomenology, narrative research) and guiding theory if appropriate; identifying the research paradigm (e.g., postpositivist, constructivist/ interpretivist) is also recommended; rationale**	4 - 5
6	**Researcher characteristics and reflexivity** - Researchers' characteristics that may influence the research, including personal attributes, qualifications/experience, relationship with participants, assumptions, and/or presuppositions; potential or actual interaction between researchers' characteristics and the research questions, approach, methods, results, and/or transferability	4
7	**Context** - Setting/site and salient contextual factors; rationale**	4
8	**Sampling strategy** - How and why research participants, documents, or events were selected; criteria for deciding when no further sampling was necessary (e.g., sampling saturation); rationale**	4 - 5
9	**Ethical issues pertaining to human subjects** - Documentation of approval by an appropriate ethics review board and participant consent, or explanation for lack thereof; other confidentiality and data security issues	11
10	**Data collection methods** - Types of data collected; details of data collection procedures including (as appropriate) start and stop dates of data collection and analysis, iterative process, triangulation of sources/methods, and modification of procedures in response to evolving study findings; rationale**	4 - 5
11	**Data collection instruments and technologies** - Description of instruments (e.g., interview guides, questionnaires) and devices (e.g., audio recorders) used for data collection; if/how the instrument(s) changed over the course of the study	4
12	**Units of study** - Number and relevant characteristics of participants, documents, or events included in the study; level of participation (could be reported in results)	Included in results
13	**Data processing** - Methods for processing data prior to and during analysis, including transcription, data entry, data management and security, verification of data integrity, data coding, and anonymization/de-identification of excerpts	4 - 5
14	**Data analysis** - Process by which inferences, themes, etc., were identified and developed, including the researchers involved in data analysis; usually references a specific paradigm or approach; rationale**	4 – 5

For peer review only

15	**Techniques to enhance trustworthiness** - Techniques to enhance trustworthiness and credibility of data analysis (e.g., member checking, audit trail, triangulation); rationale**	10
	**Results/findings**	
16	**Synthesis and interpretation** - Main findings (e.g., interpretations, inferences, and themes); might include development of a theory or model, or integration with prior research or theory	5 – 8
17	**Links to empirical data** - Evidence (e.g., quotes, field notes, text excerpts, photographs) to substantiate analytic findings	5 – 8
	**Discussion**	
18	**Integration with prior work, implications, transferability, and contribution(s) to the field -** Short summary of main findings; explanation of how findings and conclusions connect to, support, elaborate on, or challenge conclusions of earlier scholarship; discussion of scope of application/generalizability; identification of unique contribution(s) to scholarship in a discipline or field	8 – 10
19	**Limitations** - Trustworthiness and limitations of findings	10
	**Other**	
20	**Conflicts of interest** - Potential sources of influence or perceived influence on study conduct and conclusions; how these were managed	10
21	**Funding** - Sources of funding and other support; role of funders in data collection, interpretation, and reporting	11

*The authors created the SRQR by searching the literature to identify guidelines, reporting standards, and critical appraisal criteria for qualitative research; reviewing the reference lists of retrieved sources; and contacting experts to gain feedback. The SRQR aims to improve the transparency of all aspects of qualitative research by providing clear standards for reporting qualitative research.

**The rationale should briefly discuss the justification for choosing that theory, approach, method, or technique rather than other options available, the assumptions and limitations implicit in those choices, and how those choices influence study conclusions and transferability. As appropriate, the rationale for several items might be discussed together.

Citation:

O'Brien, Bridget C. PhD; Harris, Ilene B. PhD; Beckman, Thomas J. MD; Reed, Darcy A. MD, MPH; Cook, David A. MD, MHPE Standards for Reporting Qualitative Research, Academic Medicine: September 2014 - Volume 89 - Issue 9 - p 1245-1251 doi: 10.1097/ACM.0000000000000388.

For peer review only
